# General Relationship of Global Topology, Local Dynamics, and Directionality in Large-Scale Brain Networks

**DOI:** 10.1371/journal.pcbi.1004225

**Published:** 2015-04-14

**Authors:** Joon-Young Moon, UnCheol Lee, Stefanie Blain-Moraes, George A. Mashour

**Affiliations:** 1 Department of Anesthesiology, University of Michigan Medical School, Ann Arbor, Michigan, United States of America; 2 Department of Anesthesiology and Center for Consciousness Science, University of Michigan Medical School, Ann Arbor, Michigan, United States of America; 3 Department of Anesthesiology, Center for Consciousness Science and Neuroscience Graduate Program, University of Michigan Medical School, Ann Arbor, Michigan, United States of America; University of Connecticut, UNITED STATES

## Abstract

The balance of global integration and functional specialization is a critical feature of efficient brain networks, but the relationship of global topology, local node dynamics and information flow across networks has yet to be identified. One critical step in elucidating this relationship is the identification of governing principles underlying the directionality of interactions between nodes. Here, we demonstrate such principles through analytical solutions based on the phase lead/lag relationships of general oscillator models in networks. We confirm analytical results with computational simulations using general model networks and anatomical brain networks, as well as high-density electroencephalography collected from humans in the conscious and anesthetized states. Analytical, computational, and empirical results demonstrate that network nodes with more connections (i.e., higher degrees) have larger amplitudes and are directional targets (phase lag) rather than sources (phase lead). The relationship of node degree and directionality therefore appears to be a fundamental property of networks, with direct applicability to brain function. These results provide a foundation for a principled understanding of information transfer across networks and also demonstrate that changes in directionality patterns across states of human consciousness are driven by alterations of brain network topology.

## Author Summary

Current brain connectome projects are attempting to construct a map of the structural and functional network connections in the brain. One goal of these projects is to understand how network organization determines local functions and information transfer patterns, which is essential to achieve higher cognitive brain functions. Because of the limitation of constructing all brain maps for all cognitive states, finding a general relationship of global topology, local dynamics and the directionality of information transfer in a network is crucial. In this study, we show that inter-node directionality arises naturally from the topology of the network. Analytical, computational, and empirical results all demonstrate that network nodes with more connections (i.e., higher degree) lag in phase, while lower-degree nodes lead. Our mathematical analysis allowed us to predict the directionality patterns in general model networks as well as human brain networks across different states of consciousness. These findings may provide more straightforward approaches to dissecting how directionality between interacting nodes is shaped in complex brain networks, providing a foundation for understanding principles of information transfer. Furthermore, the underlying mathematical relationship between node connections and directionality patterns has the potential to advance network science across numerous disciplines.

## Introduction

Current large-scale initiatives are attempting to construct a map of the structural and functional network connections in the brain [1, 2]. One critical goal of these initiatives is to understand the mechanism by which local and functionally specialized neural activity becomes globally integrated to achieve efficient brain function [3–5]. Neural oscillations may represent one mechanism of what is sometimes referred to as “information flow” between segregated neural nodes [6–9]. However, in order to understand the principles of information transfer across networks, the mechanisms of *directionality* between the oscillations of interacting nodes need to be elucidated.

There have been a number of computational studies on the relationship of network structures, local dynamics, and directional connectivity [10–13]. More recently, a causal relationship between global brain network topology and the dynamics of corticocortical interactions has been postulated [14, 15]. Emerging empirical data and computational models suggest that the relative location of neuronal populations in large-scale brain networks might shape the neural dynamics and the directional interactions between nodes, which implies a significant influence of global topology on local dynamics and information flow [16–21]. For example, a study analyzing the electroencephalogram (EEG) recorded from human volunteers demonstrated that if a brain region is topologically more accessible to other brain regions, then it has a larger variability in its local activity [16]. As another example, a magnetoencephalogram (MEG) study showed that variability in the MEG sources determines the direction of information flow between local brain regions [17, 18]. These studies provide empirical evidence of a direct influence of brain network topology on variability of local brain activity and directionality in brain networks. In addition, computational models and simulation studies of global brain networks have revealed that hub nodes (i.e., nodes with extensive connections) have a significant influence on the local node dynamics and the direction of information flow in normal and pathological brains [19–21]. For example, Stam et al. showed in a model that the phase lead/lag relationship between local node dynamics is correlated with the degree of the node [19]. However, these past studies all describe special cases without analytical or direct empirical support; a general mechanism that links global network topology, local node dynamics and information flow has yet to be identified.

**Fig 1:**
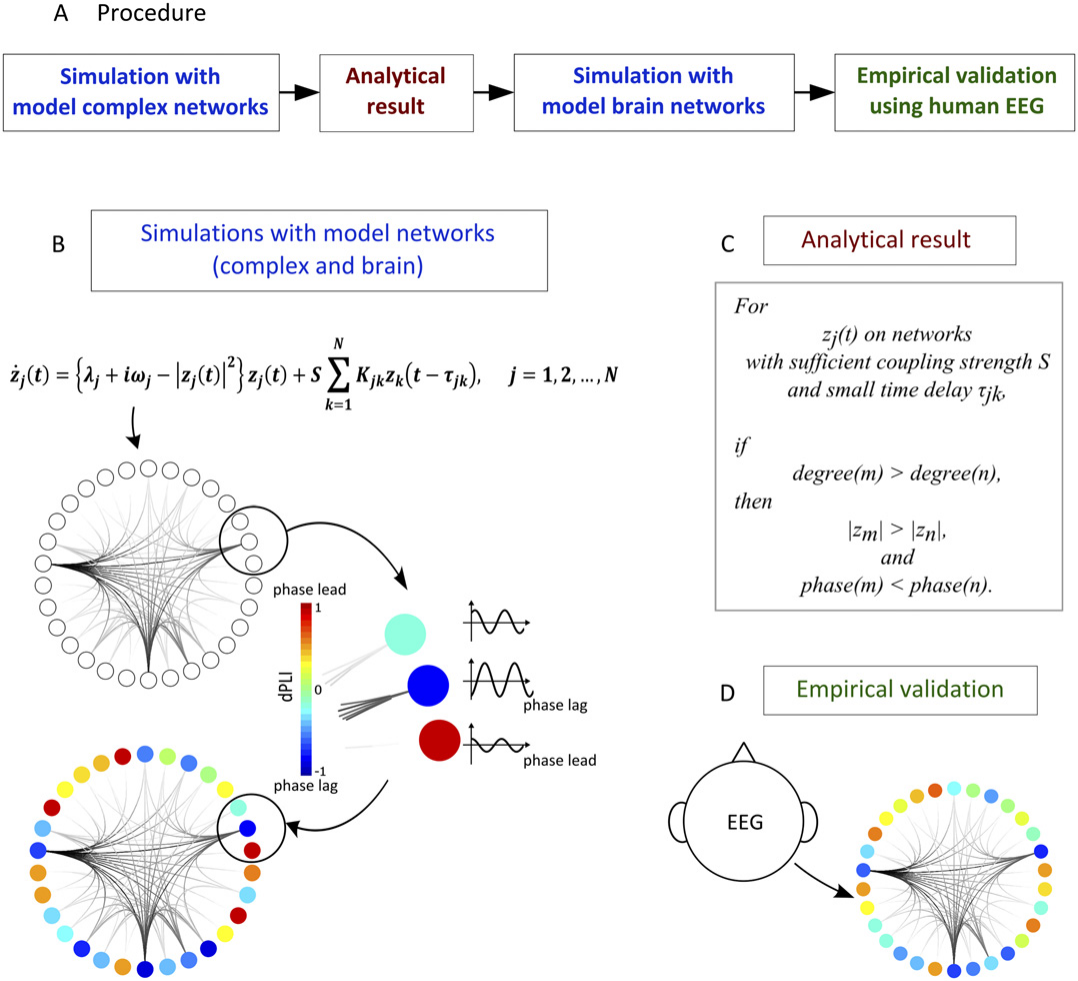
Methodological flow of the study. (A) The methodology of the study is shown sequentially. We simulated oscillators *z*_*j*_ *(t)* on model complex networks, then derived the analytical result. We applied the same simulation scheme for the human anatomic network and empirically validated the result from human EEG analysis. We made predictions by applying the simulation scheme to the human brain networks. (B) The simulation scheme for networks is shown. Stuart-Landau oscillators *z*_*j*_ *(t)* were applied to the node of each network. We measured whether the signals from each oscillator would phase lead or lag compared to other oscillators using dPLI. (C) We analytically demonstrate that for oscillators *z*_*j*_ *(t)* on networks with sufficient coupling strength *S* and small time delay *τ*_*jk*_, if degree of node m is larger than degree of node *n*, the amplitude will be larger and phase lag *n*. (D) From 64 channel human EEG data, we constructed a connectivity network between each channel and measured phase lead/lag relationships by dPLI.

In the current study we address an important prerequisite to understanding this general mechanism by identifying the relationship of topology, local dynamics and directionality. The directionality of interactions between nodes was studied through the modulated phase lead/lag relationship of coupled oscillators in general network models, large-scale anatomical brain network models and empirically-reconstructed networks from high-density human EEG across different states of consciousness (Fig 1). Analytical, computational and empirical results demonstrate definitively that the node degree (i.e., the number of connections to other nodes) defines both the directionality between local node dynamics and the amplitude of the oscillations at that node. Importantly, the directionality is shown to result from inhomogeneous interactions of local dynamics and can be differentiated from the conventional observation of directed physical connections.

## Results

### Identification of mathematical relationships among node degree, amplitude of local oscillations and directionality of interactions

The central purpose of this study was to identify a general relationship of network topology, local node dynamics and directionality in inhomogeneous networks. We proceeded by constructing a simple coupled oscillatory network model, using a Stuart-Landau model oscillator to represent the neural mass population activity at each node of the network (see Materials and Methods, and S1 Text for details). The Stuart-Landau model is the normal form of the Hopf bifurcation, which means that it is the simplest model capturing the essential features of the system near the bifurcation point [22–25]. The Hopf bifurcation appears widely in biological and chemical systems [24–33] and is often used to study oscillatory behavior and brain dynamics [25, 27, 29, 33–36].

We first ran 78 coupled Stuart-Landau models on a scale-free model network [37, 38]—that is, a network with a degree distribution following a power law—where coupling strength *S* between nodes can be varied as the control parameter. The natural frequency of each node was randomly drawn from a Gaussian distribution with the mean at 10 Hz and standard deviation of 1 Hz, simulating the alpha bandwidth (8-13Hz) of human EEG, and we systematically varied the coupling strength *S* from 0 to 50. We also varied the time delay parameter across a broad range (2~50ms), but this did not yield a qualitative difference in the simulation results as long as the delay was less than a quarter cycle (< 25 ms) of the given natural frequency (in this case, one cycle is about 100 ms since the frequency is around 10Hz). The simulation was run 1000 times for each parameter set. Subsequently, the directionality between all local node dynamics was measured using the directed phase lag index (dPLI), which calculates the phase lead and lag relationship between two oscillators (see Materials and Methods for detailed definition) [19].

dPLI between two nodes *a* and *b*, dPLI_*ab*_, can be interpreted as the time average of the sign of phase difference $\phi_{a}^{*}-\phi_{b}^{*}$. It will yield a positive/negative value if *a* is phase leading/lagging b, respectively. dPLI was used as a surrogate measure for directionality between coupled oscillators [19]. Without any initial bias, if one node leads/lags in phase and therefore has a higher/lower dPLI value than another node, the biased phases reflect the directionality of interaction of coupled local dynamics. dPLI was chosen as the measure of analysis because its simplicity facilitated the analytic derivation of the relationship between topology and directionality. However, we note that we also reach qualitatively similar conclusions with our analysis of other frequently-used measures such as Granger causality (GC) and symbolic transfer entropy (STE) (see S1 Text and S1 Fig for the comparison) [39–41].

**Fig 2:**
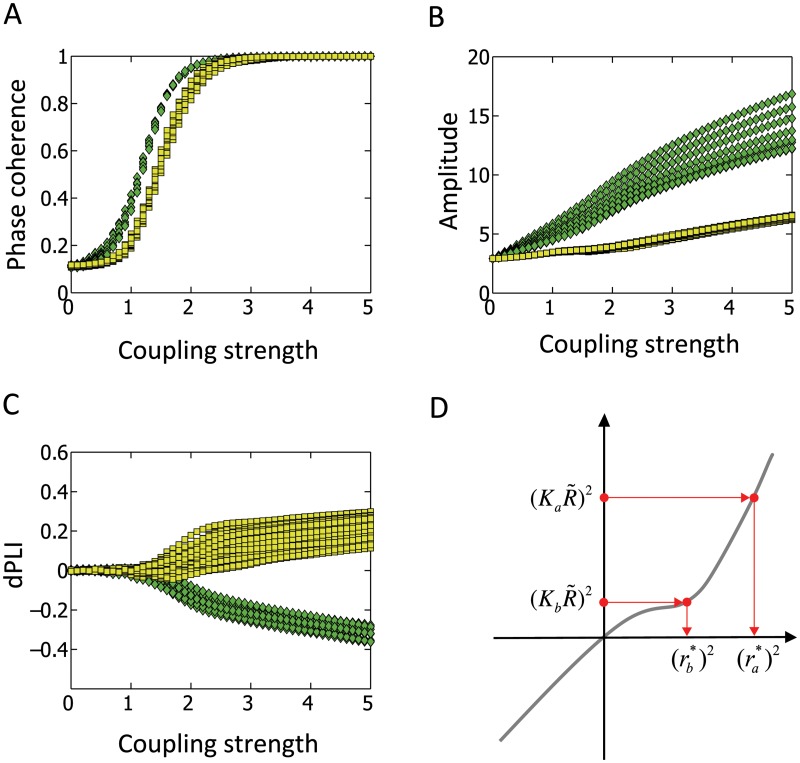
Distinct local dynamics of hub and peripheral nodes. A coupled Stuart-Landau model oscillator was simulated on a scale-free network with 78 nodes; distinct local dynamics at hub nodes (green triangle: defined as nodes with degree above the group standard deviation), and peripheral nodes (yellow circle: defined as nodes with degree 1) are found as coupling strength *S* is varied. (A) Mean phase coherence (PC), (B) amplitude, and (C) averaged dPLI for the two groups of nodes are presented. (D) The average coupling strength of *j*, *K*_*j*_, is shown as function of amplitude $\;r_{j}^{*}$. Here, $\tilde{R}$ is the order parameter. We analytically identified that ${\left(K_{j}\tilde{R}\right)}^{2}$ is a monotonic increasing function of ${\left(r_{j}^{*}\right)}^{2}$, such that $if\;K_{a}>K_{b},\;then\;\;r_{a}^{*}>r_{b}^{*}$. For the simulation, the time delay between each node was given as 10ms.

**Fig 3:**
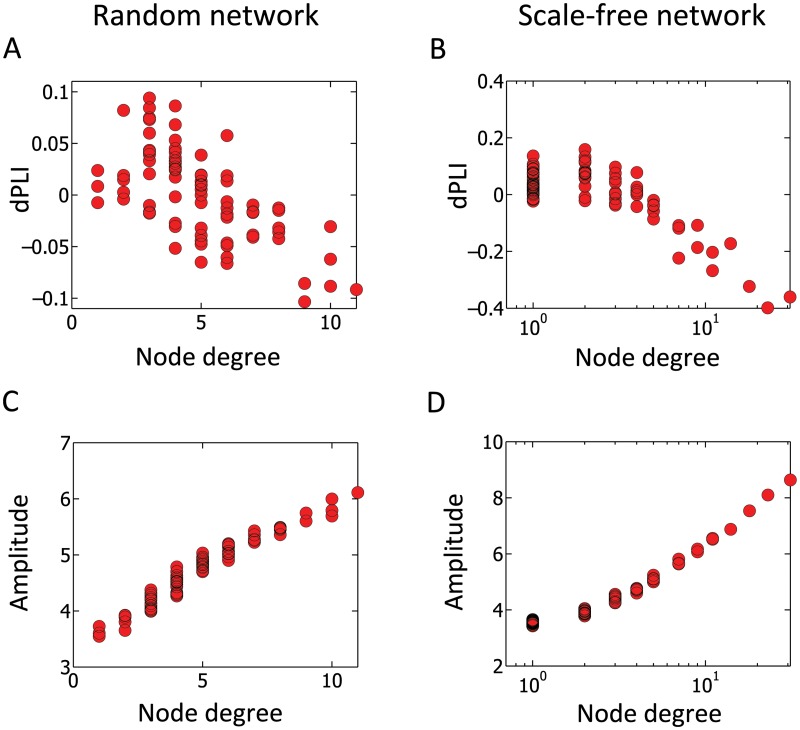
Relationships of node degree, amplitude and dPLI in inhomogeneous model networks. The Stuart-Landau model was simulated on two different inhomogeneous networks, a random network (A, C) and a scale-free network (B, D). The dPLI and amplitude have strong correlations with node degree, which demonstrate the relationship between network topology (node degree) and local node dynamics (i.e., phase and amplitude modulation). Larger node degrees have phase lag (dPLI <0) and larger amplitude, while smaller node degrees have phase lead (dPLI >0) and smaller amplitude, irrespective of the type of inhomogeneous network. Average dPLI for each node was calculated by averaging the dPLI values of each node with respect to all other nodes. For the simulation, the time delay between each node was given as 10ms. The coupling strength *S* was set to 1.5, where the separation of activities between hub nodes and peripheral nodes begins to emerge.

Fig 2A–2C demonstrates how the network topology is related to the amplitude and phase of local oscillators. Fig 2A shows the mean phase coherence (measure of how synchronized the oscillators are; see Materials and Methods for details) [42] for two groups of nodes in the network: 1) hub nodes, here defined as nodes with a degree above the group standard deviation (green triangles, 8 out of 78 nodes); and 2) peripheral nodes, here defined as nodes with a degree of 1 (yellow circles, 33 out of 78 nodes). When the coupling strength *S* is large enough, we observed distinct patterns for each group. For example, at the coupling strength of *S* = 1.5, which represents a state in between the extremes of a fully desynchronized and a fully synchronized network (with the coherence value in the vicinity of 0.5), the amplitudes of node activity are separated into two groups—hub nodes, with larger amplitudes, and peripheral nodes, with smaller amplitudes (Fig 2B). More strikingly, the phase lead/lag relationship is clearly differentiated between the hub and peripheral nodes: hub nodes phase lag with dPLI <0, while the peripheral nodes phase lead with dPLI >0 (Fig 2C). Fig 3 shows the simulation results in random and scale-free networks, which represent two extreme cases of inhomogeneous degree networks. This figure clearly demonstrates that larger degree nodes lag in phase with dPLI <0 and larger amplitude (see S2 Fig for various types of networks: scale free, random, hierarchical modular and two different human brain networks) even at the coupling strength *S* = 1.5, where the separation of activities between hub nodes and peripheral nodes just begins to emerge. To explain these simulation results, we utilized Ko et al.’s mean-field technique approach to derive the relationships for the coupled Stuart-Landau oscillators with inhomogeneous coupling strength, which in turn can be applied to inhomogeneous degree networks by interpreting inhomogeneous coupling strength as inhomogeneous degree for each oscillator [43]. We then proceeded to identify the relationships between network topology (node degree), node dynamics (amplitude) and directionality between node dynamics (dPLI) (see S1 Text for complete derivation).

The analytical results demonstrate that, for the Stuart-Landau oscillators with the same natural frequencies and inhomogeneous coupling, when the coupling strength between oscillators is sufficiently high and the delay time given as constant between them is sufficiently small, ${\left(K_{j}\tilde{R}\right)}^{2}$ can only have a monotonically increasing relationship with respect to ${\left(r_{j}^{*}\right)}^{2}$ as shown in Fig 2D. Here *K*_*j*_ corresponds to the average coupling strength to oscillator *j*, and is interpreted as the degree of node *j*, *k*_*j*_, times the coupling strength *S* (*K*_*j*_≈ *k*_*j*_ *S*), and $\tilde{R}$ is the order parameter (sum of all oscillators: see S1 Text for details). Therefore, the following relationship holds:

$$if\;K_{a}>K_{b},\;then\;r_{a}^{*}>r_{b}^{*}.$$ (1)

In other words, nodes with higher degrees naturally have larger amplitudes. The analytic results also demonstrate the following:

if ra*>rb*, then tan⁡ϕa*-Φ+β<tan⁡(ϕb*-Φ+β) (2)

Accordingly, if $\left(\phi_{a}^{*}-\Phi +\beta \right)\in \left[-\frac{\pi }{2},\;\frac{\pi }{2}\right]$, then $\phi_{a}^{*}<\phi_{b}^{*}$, for tan(*x*) is monotonically increasing function of *x* for $x\in \left[-\frac{\pi }{2},\;\frac{\pi }{2}\right]$. Here $\phi_{a}^{*}$ and $\phi_{b}^{*}$ are the phase of node *a* and *b*, respectively, Ф is the average phase across all nodes and *β* is the time delay. The inequality states that if the amplitude of node *a* is larger than that of node *b*, then it follows that the phase of node *a* is smaller than the phase of node *b*. Thus, *a* will phase lag *b*.

Therefore, given two nodes *a* and *b* with their degrees *k*_*a*_>*k*_*b*_, our results show that the amplitudes and phases will be $r_{a}^{*}>r_{b}^{*}$ and $\phi_{a}^{*}<\phi_{b}^{*}$, respectively. By definition, dPLI_*ab*_ (defined as the time average of the sign of phase difference $\phi_{a}^{*}-\phi_{b}^{*}$) will have a negative value. In short, higher-degree nodes have larger amplitude and phase lag (dPLI<0), while lower-degree nodes have smaller amplitude and phase lead (dPLI>0). The inequalities for node degree *k*, amplitude r* and phase ϕ* mathematically explain how the degree of a network node is related to the amplitude and phase of oscillation.

We note that we have also repeated the same analysis with the coupled Kuramoto model, which is the canonical model capturing the dynamics of the oscillator network with only a single phase variable for each oscillator [6, 25, 33, 44, 45] (see S1 Text for its relationship to more complex models), and found it yields the same result: higher degree nodes phase lag with dPLI <0 (see S1 Text for the analytical derivation and S3 Fig for the simulation result). In the next section, our analytic studies for two extreme cases of inhomogeneous networks of Gaussian (random) and power-law (scale-free) degree distributions will be applied to complex human brain networks.

### Confirmation of node degree/directionality relationship in a computational model of human brain networks

The network topology of the human cortex consists of primary hubs in the posterior-parietal region with most peripheral nodes located in the frontal region [46–48]. We predict that this archetypical topology gives rise to the characteristic amplitude topography and directionality pattern observed in the human brain. To test this hypothesis, we simulated human brain networks for both conscious and unconscious (i.e., uncoupled) states. An anatomical network from diffusion tensor imaging (DTI) was used as the underlying network for the model oscillators [47]. Each network node represents one of 78 cortical regions and two nodes were considered connected if the probability of fiber connections exceeded a statistical criterion. The anatomical network has the following properties: 1) small-world network, 2) scale-free degree distribution with an exponential cut-off, 3) higher degree nodes are mostly distributed in the parietal and occipital lobes, whereas the lower degree nodes are located in the frontal lobe. Alpha-band neural oscillations were simulated with 78 coupled Stuart-Landau models on the anatomical network. In order to study the effect of changing the brain network topology, we also perturbed the anatomical network in proportion to the degree of the nodes. Therefore, the hub structures were preferentially disrupted, which is consistent with empirical observations of the behavior of the human brain during anesthetic-induced unconsciousness [49].

In mathematical terms, the preferential disruption of hub nodes is given by multiplying *1/g* ^*γ*^ factor to the coupling strength *S* in eqs (2) and (3) (see Materials and Methods). Here *g* is the degree for each node, and *γ* is the perturbation strength. Higher values of *γ* generate stronger perturbations of the node. For *γ = 1*, the network becomes homogeneous with the coupling strength *S* for a node normalized by its degree: *S*/*g*. Otherwise, if *γ* >1, the coupling term *S*/*g* ^γ^ will be smaller for a node with high degree producing a larger perturbation effect for such a node. Therefore, an excessive perturbation of *γ* >>1 will yield an inverse hub-periphery structure.

**Fig 4:**
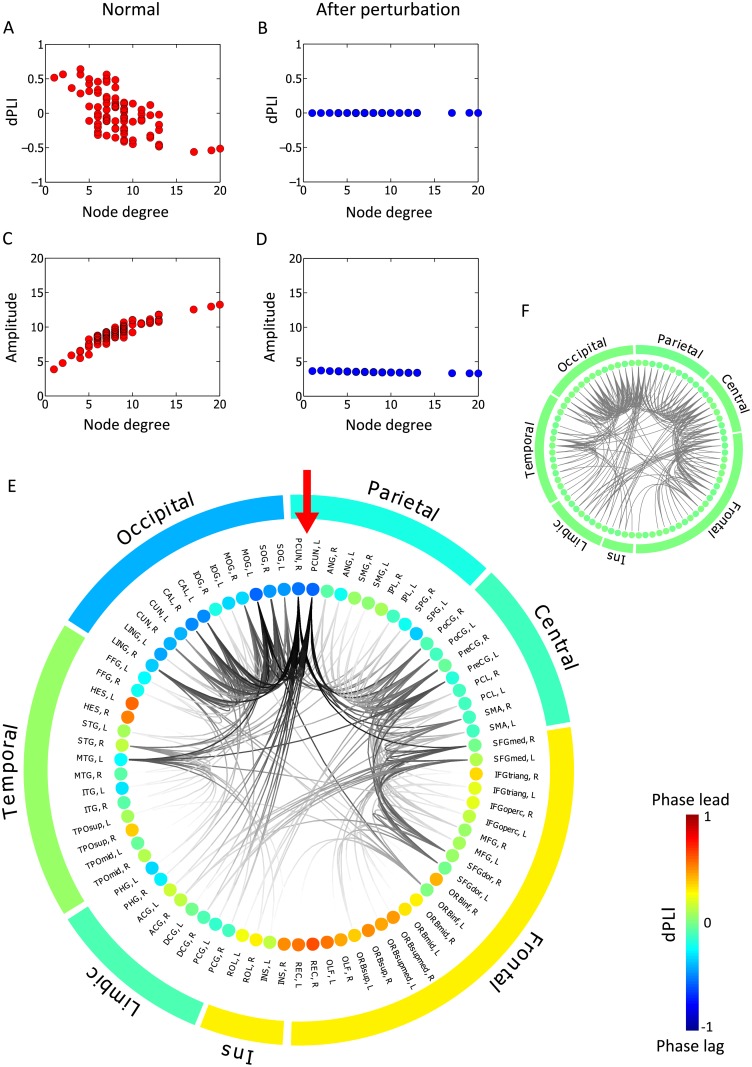
Relationships of node degree, amplitude and dPLI in human neuroanatomical networks. The Stuart-Landau model was simulated on the human anatomical brain network before ((A), (C) and (E)) and after ((B), (D) and (F)) perturbation with preferential disruption of hub nodes. The general relationship of node degree, amplitude and dPLI is also demonstrated in this modeled human brain network. The strong negative correlations between node degree and dPLI in (A) and the strong positive correlation between node degree and amplitude in (C) disappear in the perturbed homogeneous network ((B) and (D)). Average dPLI for each node was calculated by averaging the dPLI values of each node with respect to all other nodes. The anatomical connectivity of different brain regions are presented in (E) and (F) ring plots together with average dPLI value for each region. The nodes are aligned in groups: frontal lobe, central regions (including motor and somatosensory cortex), parietal lobe, occipital lobe, temporal lobe, limbic region, and Insula (Ins). Red arrow in (E) points to left and right precuneus. Color of each node shows the average dPLI values with respect to other nodes, from red (dPLI = 1) to blue (dPLI = -1). Average dPLI for each group is also shown in color. The inset within the ringplot shows connections between nodes, highlighted by darker color if the node has a higher degree of connections. Only the links from hub nodes (node with degree value within top 30%) are colored. In the simulation, the time delay between each node was given proportional to the delay, with propagation speed of 6m/s. The coupling strength *S* was given as 3. The full names for the cortical regions of the human brain network are available in Gong et al. [47].

Fig 4A and 4C clearly demonstrate a negative correlation between node degree and dPLI (Spearman correlation coefficient = - 0.61, p<0.01) and positive correlation between node degree and amplitude of oscillators (Spearman correlation coefficient = 0.92, p<0.01) at coupling strength *S* = 3. As predicted, higher degree nodes have higher amplitude and stronger incoming directionality than lower degree nodes (dPLI<0). Fig 4B and 4D show that after the perturbation (*γ = 1*), the correlations among node degree, amplitude and dPLI disappear. The homogenized network does not produce any biases in the directionality and amplitude distribution in the modeled brain. Fig 4E and 4F present the relationship between the node degree of the anatomical brain network and dPLI of the alpha oscillators as a ring plot. In the anatomical brain network, the parietal-occipital regions have the higher node degrees (presented as dense and dark connections). Notably, the left and right precuneus in the parietal region have the highest node degrees (denoted with red arrows in Fig 4E), while the lower node degrees are mostly distributed in the frontal region. The functional network strongly correlates with the anatomical network. Accordingly, the two precuneus regions have the largest negative dPLI values, playing a role as the strongest target of directionality, and the typical overall network topology produces the dominant directionality from frontal region (as source; red color (dPLI>0) in the ring in Fig 4E) to the parietal-occipital region (as sink; blue color (dPLI<0) in the ring in Fig 4E). However, after perturbing the heterogeneous human network to a homogeneous functional network topology, the typical patterns in amplitude and directionality are neutralized (presented as the same green color (dPLI~0) in the ring in Fig 4F).

In summary, this simulation of normal and perturbed human brain networks clearly demonstrates that the typical topology of the anatomical brain network shapes the spatial distribution of node amplitude and the characteristic directionality patterns. Furthermore, the perturbed network topology with preferential hub disruption produces homogenized patterns in amplitude and directionality across the network. To test whether or not these results depend on the given network, we repeated the same analysis with another human anatomical network, which is based on 66 parcels of the cerebral cortex [46], and observed qualitatively similar results (see S4 Fig).

### Confirmation of node degree/directionality relationship in human EEG networks during conscious and unconscious states

**Fig 5:**
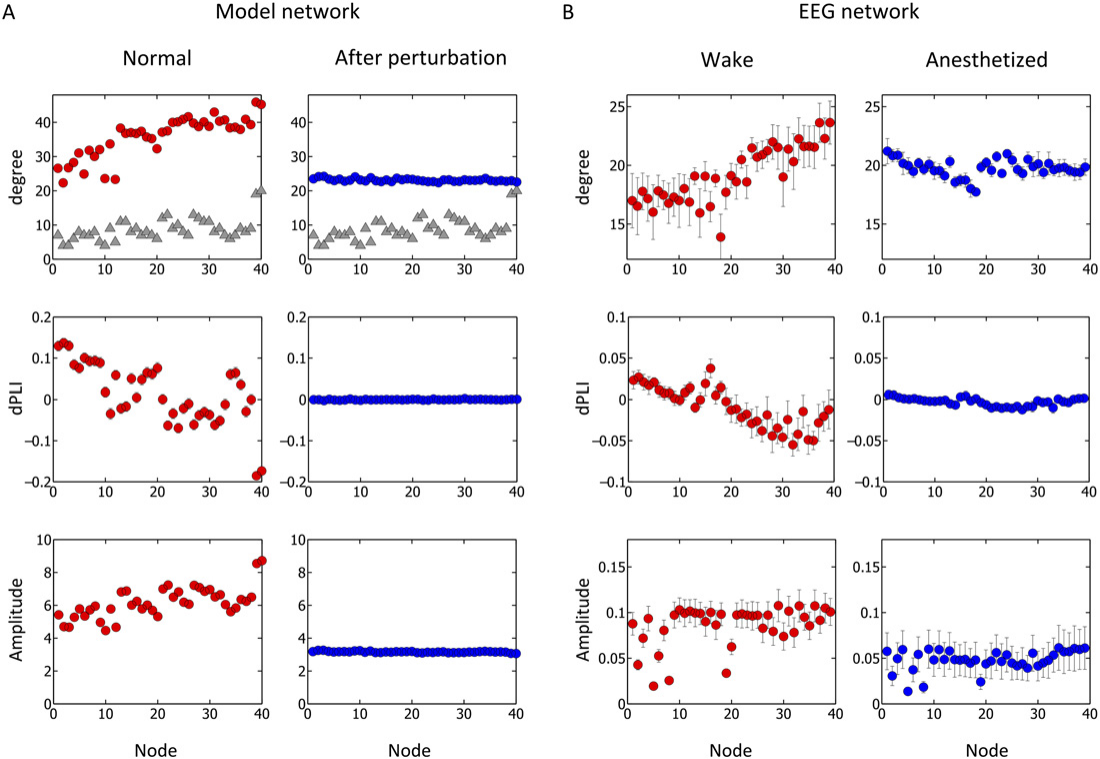
Comparison between model network and EEG network. Results were compared from the Stuart-Landau model on the human anatomical brain network of Gong et al. (A) and functional networks reconstructed from EEG (B). For each case, degree, average dPLI, and amplitude for each node is plotted with standard error, before and after perturbing the anatomic network for (A), and in wake (eyes closed) and anesthetized states for (B). In the graphs of the first row, the degree of nodes in red/blue circle is from PLI of the functional network constructed for each case. For the case of the model, (A), the degree of anatomical network is also shown in gray triangle for comparison. The nodes are aligned in a way that they are grouped by regions and span from frontal lobe to parietal lobe. For (A), nodes 1~22 are from frontal lobe, nodes 23~30 are from central regions (motor and somatosensory cortex), and nodes 31~40 are from parietal lobe. For (B), nodes 1~18 are from frontal lobe, nodes 19~29 are from central regions, and nodes 31~39 are from parietal lobe. For the simulation, time delay between each node was given proportional to the delay, with propagation speed of 6m/s. The coupling strength *S* was given as 1.5.

In order to verify the theoretical predictions of the directionality and amplitude patterns in human brain networks before and after perturbation, we analyzed empirical EEG data collected from human volunteers in states of consciousness (eyes closed, at rest) and anesthetic-induced unconsciousness. Since anesthesia primarily disrupts hub structures in the human brain network [49], we predicted that the directionality toward the hub nodes would be preferentially disrupted, which would manifest in the empirical data as a disruption of front-to-back directionality between primary peripheral nodes in frontal region and primary hub nodes in posterior-parietal regions. 64-channel EEG was recorded continuously from 7 healthy human volunteers during consciousness and sevoflurane-induced unconsciousness; 5-minute artifact-free epochs were analyzed (see Materials and Methods for the details on the EEG experiment). Recorded data were referenced to the vertex. After the experiment, EEG data were re-referenced to an average reference, and data from the vertex channel was calculated, yielding a total of 65 EEG data channels for analysis. Graph theoretic network analysis was applied to construct functional brain networks from the EEG. Phase lag index (PLI), a measure of phase locking between two signals, was calculated between all combinations of EEG channels, and channel pairs constituting the top 30% of PLI values, a threshold at which the results match well with those of model network, were chosen as the functional connections of the network [50]. The directionality was estimated for each channel by calculating the average dPLI between a given channel and each of the remaining 64 EEG channels. Because anesthesia causes a large spectral change in EEG during the transition from consciousness to unconsciousness, we examined 6 frequency bands (delta: 0.5–4Hz, theta: 4–8Hz, alpha: 8–13 Hz, beta: 13–25Hz, gamma: 25–55Hz and the whole band: 0.5–55Hz) and their respective functional networks. Our analysis demonstrated that: (1) the theoretical predictions made from computational human brain models regarding the relationship between node degree and dPLI are supported by patterns observed in empirical EEG networks recorded from waking and unconscious states (in Fig 5A and 5B); (2) The functional brain network of the whole frequency band (0.5–55Hz) is highly correlated with the node degree distribution found in the anatomical brain network model. The majority of hub nodes were located in the posterior-parietal region in both the anatomical network and the functional EEG network. In the waking state, the high-degree nodes were mainly distributed in the back part of the brain (upper row in Fig 5B), while in the unconscious state, this pattern was completely disrupted (upper row in Fig 5B); (3) The alpha band (8–13HZ) EEG network that has been the focus of our computational simulations demonstrates a dominant front-to-back directionality in the brain during the conscious state (eyes closed), with frontal dPLI > 0 and posterior dPLI < 0 (the 2nd row in Fig 5B) [51, 52], which was neutralized in the unconscious state. This neutralized directionality in the EEG network supports the results of our simulation in which we preferentially perturbed hub nodes (the 3rd row in Fig 5A and 5B); (4) The correlation between node degree (of the whole band, 0.5–55Hz) and directionality (of the alpha band, 8–13HZ) changes significantly across states. The strong negative correlation observed during the conscious state (Spearman correlation coefficient of -0.76 (p<0.01)) disappears during the unconscious state (Spearman correlation coefficient of -0.04 (p<0.01)). These correlations are consistent with the theoretical predictions from the analytical solution and simulations. However, the correlation between node degree and amplitude for the EEG network differs from the models (non-significant Spearman correlation coefficient of 0.266 (p = 0.1) for the conscious state). The lack of significance is potentially due to a distortion of the scalp EEG recording as the signals pass through the skull, which may cause a deviation from the model prediction. MEG would be more appropriate to study the correlation of amplitude and node degree in the whole brain network.

## Discussion

In this study, we provide a general relationship for how network topology (node degree) determines the directionality (phase lead/lag relationship) and local dynamics (amplitude of oscillator) using the mean-field approximation. Simple oscillatory models (Kuramoto/Stuart-Landau models) were used first to simulate the global network dynamics and to find the mathematical relationship among node degree, local dynamics and directionality (defined by phase lead/lag). We have shown that the directionality arises naturally from the topology of the underlying network. The hub nodes phase lag: they act as a sink that is driven by connected nodes. The non-hub peripheral nodes phase lead: they are sources and drive the connected nodes. This finding may be counterintuitive, as network hubs could be regarded as “control centers” that serve as the source of outflowing information. The present results suggest, by contrast, that hub nodes with high degree may “attract” information from peripheral nodes. The consistently phase-lagging nature of the high-degree hub node may allow for the inputs of spatially and functionally distinct peripheral nodes to converge and be integrated, a critical feature for optimal network function. Network topology also predicts the local dynamics, defined here by the amplitude of an oscillation in the case of the Stuart-Landau model; high degree hub nodes are associated with oscillations of larger amplitude and low degree peripheral nodes are associated with oscillations of smaller amplitude.

There have been several important studies exploring the effect of brain network topology on the local and global dynamics of the brain. De Haan et al. simulated normal and diseased brain activities based on a neural mass model of the anatomical network. They found that the hub regions are associated with the highest level of activity and that excessive neuronal activity at the hub may lead to degeneration in Alzheimer’s disease [20]. Stam et al. simulated how network structure affects the phase lead/lag relationship between brain regions in a realistic brain network model [19]. Nicosia et al. showed in a network model that if two nodes are symmetrically located within a given network topology, the dynamics of the nodes will be fully synchronized even at a significant distance [53]. Angelini et al. measured Granger causality for the Kuramoto model on networks and demonstrated that inflow/outflow ratio changes depend on the degree of each node [54]. However, despite these recent empirical and computational model studies, there has been no general explanatory mechanism linking global topology, local node dynamics and directionality between interacting nodes based on mathematical derivation.

The strength of our analysis lies in its simplicity and generality. The models we employed are simple enough to analyze extensively yet succeed in capturing the essential features of dynamic behavior of the network related to the emergence of directionality. More complex models are difficult to analyze due to the abundance of equations and parameters, rendering analytic solutions difficult except for very special cases. The models used in this study are rich enough in their behavior yet simple enough to analyze and analytically calculate. Another advantage is the generality of the models: they are representative of many other oscillating systems so that the results from these models will be widely applicable. Furthermore, the analytical results are independent of the type of network, as long as the network is inhomogeneous in terms of connections. Expressing the central relationship quantitatively, when coupling strength *S* between oscillators is sufficiently weak, any system of interacting oscillators can be considered to interact only with its phases, and the Kuramoto model is the first-order approximation for such phase-only interacting oscillators. When the coupling term is stronger so that the amplitude equations must be considered, the Stuart-Landau model equation holds its generality because it is the normal form of the Hopf bifurcation. The Hopf bifurcation is one of the most frequently appearing mechanisms in models generating oscillatory behavior, as in the case of the Wilson-Cowan model, the Fitzhugh-Nagumo model and the Morris-Lecar model, among other numerous examples. One can gain general insights about the behavior of more complex interacting oscillator models by analyzing such generalized models.

Assertions regarding the applicability of findings derived from these simple models are substantiated by a number of successful predictions. First, we simulated the oscillator models in a human anatomical network and demonstrated that anterior-to-posterior directionality arises due to a network structure in which posterior regions contain more hub nodes than anterior regions. This simulated result was confirmed with empirically-reconstructed human brain networks derived from high-density EEG recordings, demonstrating again that the anterior-to-posterior directionality occurs because of the posterior-hub structure. When this hub structure is perturbed, the directionality was eliminated in the model on the simulated neuroanatomical network. When consciousness was lost after administration of the anesthetic sevoflurane in human volunteers, anterior-to-posterior directionality was similarly eliminated with the disappearance of the posterior-hub structure. Application of this principle could have relevance to clinical conditions in which hub structure may be damaged or dramatically reorganized. Altered information flow has been reported in network-altering conditions such as Alzheimer’s disease, schizophrenia, and epilepsy [5, 20, 55, 56]. Our findings not only explain why information flow changes across different brain states, but also could ultimately contribute to treating such disorders by modulating the directionality of node interactions using brain stimulation techniques.

There are a number of limitations to this study. The first relates to the relationship of phase lead/lag measures and information flow. Although it can be asserted that causal events lead and resultant effects lag (simply by virtue of the temporal constraints on cause-effect relationships), the converse assertion that every lead/lag relationship reflects a causal influence does not hold. In other words, an appropriate phase lead/lag relationship is a necessary but not sufficient condition for the kinds of interactions that are associated with information transfer. As such, we have conducted parallel analyses with other measures (GC and STE) based on distinct theoretical frameworks (linear regression and information theory, respectively) (S1 Fig). These metrics were found to parallel the measure of dPLI, thus supporting the general interpretation that our studies of directionality may, indeed, provide a foundation for future studies related to information transfer in the brain. The second limitation is that our analysis focused primarily on the 10 Hz oscillation, but this reflects our choice to investigate brain networks. The analysis can easily be applied to other frequency bandwidths, yielding similar results as long as the time delay is sufficiently small compared to the time of one oscillation. Third, our results do not reflect short-term stationary brain network behavior such as metastability [57]. Furthermore, in a long-term time scale, the brain network structure itself will change via mutual influences between network topology and local dynamics as the brain matures [58]. The time scale of our study lies in between these two extreme limits, where the functional connectivity can reflect underlying structural connectivity yet the effect of local dynamics on the network structure can be disregarded. Fourth, the Kuramoto and Stuart-Landau models are the normal forms of complex oscillator models. Thus, the results of the coupled oscillator networks—as well as the data from our EEG experiments—describe large-scale temporal and spatial behavior, i.e., network dynamics that are relatively long-term and macroscopic. As such, our simple models and the analytical results may not explain fine-scale neuronal firing relationships and the short-term dynamics of complex local connections such as the influence of a common source with different time delays. Further work is warranted to test whether the current findings apply to finer-scale dynamics. Fifth, in the empirical data test, we analyzed a functional brain network reconstructed from scalp EEG, which reflects the anatomical brain network with less spatial fidelity than the simulated network [13, 59]. Therefore, instead of examining the one-to-one correspondence between the functional networks of the empirical data and of the model, we investigated the correlation patterns among node degree, amplitude and dPLI in the EEG network and the model network. Finally, we used a simple exponential function to achieve a preferential disruption of hub structure in the simulation of anesthetic effects on the brain network. The study of more realistic perturbation functions would be an interesting future investigation to simulate diverse anesthetic effects in the brain.

In conclusion, the topological property of node degree determines local dynamics such as the amplitude of an oscillation, as well as directionality between interacting nodes. This relationship, derived from simple oscillator models, was applied successfully to complex brain network models generated computationally or reconstructed empirically. The high-degree/high-inflow relationship predicted the behavior of human brain networks across multiple states of consciousness. These findings may provide clarity to future studies of information transfer as the complexity of the human brain connectome becomes more fully elucidated. Furthermore, the analytical mechanism provided and general relationships identified have the potential to advance network science across numerous disciplines.

## Materials and Methods

### Stuart-Landau model

In order to study the general relationships among topology, node amplitude and directionality between interacting nodes in a network, we used a simple oscillatory model, the Stuart-Landau model. The Stuart-Landau model is defined as the following:

$${\dot{z}}_{j}\left(t\right)\;=\;\left\{\lambda_{j}+i\omega_{j}-{\left|z_{j}\left(t\right)\right|}^{2}\right\}z_{j}\left(t\right)+S\sum_{k\;=\;1}^{N}K_{jk}z_{k}(t-\tau_{jk}),\;j\;=\;1,2,\ldots ,N.$$ (3)

Here, z_*j*_ *(t)* is the complex variable describing the state of *j*th oscillator. The eq (3) can be separated into two variables:

$${\dot{r}}_{j}\left(t\right)\;=\;\left\{\lambda_{j}-{\left|r_{j}\left(t\right)\right|}^{2}\right\}r_{j}\left(t\right)+S\sum_{k\;=\;1}^{N}K_{jk}r_{k}\cos\left(\theta_{k}\left(t-\tau_{jk}\right)-\theta_{j}\left(t\right)\right)\;,$$ (4)

$${\dot{\theta }}_{j}\left(t\right)\;=\;\omega_{j}+S\sum_{k\;=\;1}^{N}K_{jk}\frac{r_{k}}{r_{j}}\sin\left(\theta_{k}\left(t-\tau_{jk}\right)-\theta_{j}\left(t\right)\right)\;,\;j\;=\;1,2,\ldots ,N.$$ (5)

*r*_*j*_ *(t)* is the amplitude of the signal the oscillator *j* produces at time *t*. *λ*_*j*_ *(t)* is a parameter governing the amplitude and we set all *λ*_*j*_ *(t)* for *j = 1*,*2*,*…*,*N* equal for our simulations so that the differences in the amplitude between oscillators can only come from the coupling term in each equation. Also, we note that when all the amplitudes are set equal to each other and do not change, the eqs (4) and (5) reduce to the phase-only equation, which is the Kuramoto model. In this respect the Stuart-Landau model can be considered as the generalized model of the Kuramoto model, with the inclusion of the amplitude equation. Descriptions of the Kuramoto model itself, the relationship between the Kuramoto model, the Stuart-Landau model and more complex neural mass models, and the derivation from Wilson-Cowan model to Stuart-Landau/Kuramoto models are included in the S1 Text.

For the functional connection in the network, we use two types of phase coherence measures; (1) mean phase coherence (PC) and (2) phase lag index (PLI). PC is a measure of mean phase synchronization, which can be directly calculated from the model oscillators’ phases. On the contrary, PLI measures nonzero phase lead/lag relationships, which mitigates the effects of choice of reference and of volume conduction in EEG analysis.

### Mean phase coherence (PC)

The mean phase coherence between two oscillators *j* and *k* in a network is defined as:

$${PC}_{jk}\;=\;\left|\frac{1}{T}\sum_{t\;=\;1}^{T}e^{i{\Delta \theta }_{jk}\left(t\right)}\right|,\;$$ (6)

where Δθ_*jk*_(t) is the phase difference. For complete phase synchronization, *PC*_*jk*_ has 1, and 0 for completely desynchronized case [42]. For each node *j*, we can calculate PC_*j*_ as the averaged value of *PC*_*jk*_ for all other nodes *k*. Such averaged mean phase coherence for each hub/non-hub node with respect to all other nodes in the coupled Stuart-Landau oscillator network is demonstrated in Fig 2A.

### Phase lag index (PLI)

PLI was used to define the functional connectivity in the EEG network [50]. We use a Hilbert transform to extract the instantaneous phase of the electroencephalogram from each channel and calculate the phase difference Δθ_*jk*_(t) between channels *i* and *j*, where Δθ_*ij*_(t) = θ_*i*_(t)-θ_*j*_(t), t = 1,2,…n, *n* is the number of samples within one epoch. PLI_*ij*_ between two nodes *i* and *j* is then calculated using eq (7):

$$<Display\_Math>{PLI}_{ij}\;=\;\left|\;\langle \;sign\left({\Delta \theta }_{ij}(t)\right)\;\rangle \;\right|,\;0\;\leq \;{PLI}_{ij}\;\leq 1.$$ (7)

Here, the sign() function yields: 1 if Δθ_*ij*_(t)>0; 0 if Δθ_*ij*_(t) = 0; and -1 if Δθ_*ij*_(t)<0. The mean < > is taken over all t = 1,2,…,n. If the instantaneous phase of one signal is consistently ahead of the other signal, the phases are considered locked, and PLI_*ij*_ ≈ 1. However, if the signals randomly alternate between a phase lead and a phase lag relationship, there is no phase locking and PLI_*ij*_ ≈ 0.

### Directed phase lag index (dPLI)

To determine the phase-lead/phase-lag relationship between channels, we calculate dPLI between nodes *i* and *j* using eq (8) [19]:

$${dPLI}_{ij}\;=\;\langle sign\left({\Delta \theta }_{ij}(t)\right)\rangle ,\;-1\;\leq \;d{PLI}_{ij}\;\leq 1.$$ (8)

Here, again the sign() function yields: 1 if Δθ_*ij*_(t)>0; 0 if Δθ_*ij*_(t) = 0; and -1 if Δθ_*ij*_(t)<0. The mean < > is again taken over all t = 1,2,…,n. Therefore, if on average, node *i* leads node *j*, 0< dPLI_*ij*_ ≤1; if node *j* leads node *i*, -1≤ dPLI_*ij*_ <0; and if there is no phase-lead/phase-lag relationship between nodes, dPLI = 0. In this study, dPLI_*i*_ for a node *i* can be defined as the average of dPLI_*ij*_ for all other nodes *j*. For the purpose of brevity, each time we denote dPLI of a node *i* in the Results section, we are referring to dPLI_*i*_ for the node *i*.

### Coupled Stuart-Landau/Kuramoto model parameters

All the parameters for the models are set accordingly to simulate alpha oscillations in the brain. For both models, the natural frequencies of the oscillators in our simulation are given as a Gaussian distribution to simulate alpha with mean at 10 Hz and standard deviation 1, making *ω*_*j*_ around 10∙2π rad/s. Time delay is (a) given an identical value between 2ms and 50 ms for all edges (for model networks as well as Gong et al.’s and Hagmann et al.’s human brain networks), or (b) given proportional to the physical distances for each edges with propagation speed of between 5 to 10m/s (for Gong et al.’s human brain network) [60, 61]. In the simulation, however, the difference in the propagation speed or time delay does not provide any qualitative differences in the results, as long as the resulting time delay is less than the time of a quarter cycle for the natural frequency (in the simulation, the time for one cycle is 100 ms for the given frequency of 10 Hz, thus it is less than 25 ms). The coupling strength between the oscillators is increased from 0 to 50. For the Stuart-Landau model, the amplitude parameter *λ*_*j*_ is given identically for all oscillators with a value of 2. For all simulations, we also added a Gaussian white noise *ξ*_*j*_ *(t)* of vanishing mean and standard deviation of 2 to each oscillator's equation to test the robustness of our results against random fluctuations.

### Model time series for the measurement

With each model, we produce a times series of length 10,000 for each run of the simulation, and then take the latter half of the time series for the measurement. The sampling rate of the time series is 1,000Hz, making the length of the produced time series 10s containing approximately 100 cycles of oscillation. For a given parameter set, measurement is averaged over at least 1,000 runs of the simulation. For the simulations on the random networks and the scale-free networks, a new network is generated for each run.

### Perturbation of the network

To test the role of hub structure on the node amplitude and directionality between interacting nodes, we perturbed the topology of human brain network by preferentially disrupting hub structures. The perturbation factor *1/g* ^*γ*^ is multiplied to the coupling strength *S* in eqs (4) and (5):

$${\dot{r}}_{j}\left(t\right)\;=\;\left\{\lambda_{j}-{\left|r_{j}\left(t\right)\right|}^{2}\right\}r_{j}\left(t\right)+\frac{S}{g^{\gamma }}\sum_{k\;=\;1}^{N}K_{jk}r_{k}\cos\left(\theta_{k}\left(t-\tau_{jk}\right)-\theta_{j}\left(t\right)\right)\;,$$ (9)

$${\dot{\theta }}_{j}\left(t\right)\;=\;\omega_{j}+\frac{S}{g^{\gamma }}\sum_{k\;=\;1}^{N}K_{jk}\frac{r_{k}}{r_{j}}\sin\left(\theta_{k}\left(t-\tau_{jk}\right)-\theta_{j}\left(t\right)\right)\;,\;j\;=\;1,2,\ldots ,N.$$ (10)

Here *g* is the degree for each node and *γ* is the perturbation factor. By multiplying *1/g* ^*γ*^, the effective coupling strength *S/g* ^*γ*^ depends on the degree of each node. Thus, the higher the value *γ* is, the stronger the perturbation of the hub. For *γ =* 1, the coupling term in each equation is normalized with respect to the degree of the node, thus the network topology become homogeneous. Consequently, it does not provide any asymmetric dynamics between the hub and peripheral nodes. If *γ* >1, the original hub nodes are excessively perturbed such that the original hub-periphery relations are reversed.

### Random and scale-free networks

Oscillator models were run over both random and scale-free networks with size 78, 100, and 1000, respectively. We used the Gilbert algorithm for producing a random network with the parameter of *G(N*, *(1+ε)log(N)/N)*, where *N* is the number of nodes, and *ε* is an arbitrary small number, such that the resulting network is connected. We use Catanzaro et al.’s algorithm to make a randomly connected network with scale-free node degree distribution given a priori [62]. The slope of the degree distribution was set to -2.2. The size of the network does not result in qualitative differences.

### Anatomical brain networks

The human brain network was constructed from diffusion tensor imaging (DTI) of 80 young adults [47]. The network is consisted of 78 parcels of the cerebral cortex. Another human brain network by Hagmann et al. [46], which is based on 66 parcels of the cerebral cortex, was used for the simulation, with qualitatively similar results.

### Human EEG recording during brain network modulation by general anesthesia

#### Ethics statement

The Human EEG recording was conducted at the University of Michigan Medical School and was approved by the Institutional Board Review (HUM00061087); written consent was obtained from all participants after a careful discussion of risks and benefits.

After IRB approval and written informed consent, EEG data were recorded from seven healthy volunteers (4 males, 20–23 years of age) in a conscious state with their eyes closed or a state of sevoflurane-induced unconsciousness. Sevoflurane concentration was titrated upwards in a stepwise fashion until consciousness was lost, as evidenced by cessation of following a verbal command. EEG was acquired using a 64-channel sensor net from Electrical Geodesics Inc with a sampling frequency of 500 Hz. All channels were referenced to the vertex with electrical impedance reduced to below 50 KΩ prior to data collection. After the data were collected, EEG signals were highpass filtered at 0.1 Hz, and re-referenced to an average reference. Subsequently, signals were visually inspected to reject epochs containing non-physiological artifacts. These data were gathered for a prior study and were re-analyzed here with different techniques and different hypotheses [63].

### Human EEG network analysis

The node degree, amplitude and dPLI for each node were calculated in EEG networks constructed from a 64-channel EEG dataset. First, each 5 min epoch of EEG data for both states (waking and anesthetic-induced unconsciousness) was segmented into 10 sec epochs for pseudo-stationary state. The node degree, amplitude and dPLI for individual are the averages over all the segmented data. For each segmented dataset, the band pass filter was applied for the six frequency bands. Band-pass filtering with the fifth-order Butterworth filter was applied to EEG forward and backward, correcting the potential phase shifting after band-pass filtering (“butterworth.m”, and “filtfilt.m” in Matlab; MathWorks, Natick, MA). For each frequency band, the PLI was calculated for all pairs of EEG channels and the adjacency matrix was constructed with the top 30% of PLI connections through searching for the best-fit to the simulation and robust threshold. Node degree for each channel was computed from the binary network, which counts the number of links connected to a node. The amplitude was calculated from mean power spectrum density. For power spectrum density, a Hamming window and a modified periodogram were used for each 10 sec EEG segment (in “pwelch.m”, in Matlab). dPLI for a channel was computed with averaged dPLI between the given channel and the other all EEG channels. Consequently, for a 5 min long EEG epoch, we can have the node degrees, amplitudes, and dPLIs for all 64 EEG channels. The spearman correlation coefficient was used for evaluating the correlations among node degree, amplitude and dPLI of the 64 channels (“corr.m” in Matlab).

### Synopsis of analytical derivation

The results from S1 Text can be summarized as follows: for Kuramoto oscillators and Stuart-Landau oscillators with inhomogeneous coupling strength between them, the oscillators with larger average coupling strength phase lag behind those with smaller average coupling strength, given the same natural frequencies, small enough constant time delays and sufficiently strong coupling strengths between them. For Stuart-Landau oscillators, we also show that the oscillators with larger average coupling strength have larger amplitude oscillations. We utilized Ko et al.’s mean-field technique to derive these results, and applied them to inhomogeneous degree networks as an approximation: the inhomogeneous coupling strength of each oscillator was interpreted as the inhomogeneous degree of each oscillator [43]. For simulations, we expanded our conditions further: we used a Gaussian distribution for natural frequencies of the oscillators and distance-varying time delays between the oscillators for Gong’s anatomical network. We also added a Gaussian-noise to each oscillator’s equation to test the robustness. The simulation results confirmed that the central relationship of degree, node dynamics and directionality (i.e., higher degree nodes have larger amplitudes and phase lag behind lower degree nodes) still holds firmly.

## Supporting Information

S1 Text Detailed derivations of the analytical results for “General Relationship of Global Topology, Local Dynamics, and Directionality in Large-Scale Brain Networks.” (PDF)

S1 Fig Comparison of three measures of directed connectivity using the Stuart-Landau model. The Stuart-Landau model was implemented on Gong et al.'s human brain network, and the causal relationship between network nodes was measured using (A) directed phase lag index (dPLI), (B) Granger causality (GC), (C) symbolic transfer entropy (STE). For each measure, the mean values for each node with respect to all other nodes are shown. The nodes of the network are indexed in decreasing order of the variable of interest, which is represented in color (blue representing lower values and red representing higher values) as their coupling strength changes from 0 to 5. dPLI measures phase-lead/lag relationship, GC is a surrogate measure for causality between given nodes, and STE is a surrogate for information transfer between two nodes. All three measures yield the same pattern: the higher degree nodes have more "information" transferred to them, and vice-versa. The simulation results suggest that the phase-lead/lag relation, causality, and information flow transfer are possibly all correlated with each other. For Gong et al.'s network, the delay time between two nodes were given proportional to distances between them with propagation speed of 6 m/s. All measures were performed 10 times and averaged. For GC, the model order for each measurement was chosen as 12. For STE, the embedding dimension was set to 3, and prediction time for each measurement was chosen to yield the maximal possible value of STE.
(EPS)

S2 Fig Distinct local dynamics of hub and peripheral nodes for different networks. A coupled Stuart-Landau model oscillator was simulated on (A) a scale-free network, (B) a random network (C) a hierarchical modular network of Sales-Pardo et al., (D) a human brain network of Gong et al., and (E) a human brain network of Hagmann et al. Each plot shows distinct local dynamics for hub nodes (darker green diamond: defined as nodes with degree above the group standard deviation), and peripheral nodes (lighter green square: defined as nodes with degree 1 for scale-free network, and as nodes with degree below the group standard deviation for other networks) as coupling strength S is varied. Mean phase coherence (PC), amplitude, and averaged directed phase lag index (dPLI) for the two groups of nodes are presented for each network. For Gong et al.'s network, delay time between two nodes was given proportional to distances between them with propagation speed of 6 m/s. For random, scale-free, hierarchical modular and Hagmann et al.'s network the delay was assigned a value of 10ms. All simulations were performed 1000 times and averaged, and new random, scale-free, and hierarchical modular network were generated with each simulation performed.
(EPS)

S3 Fig Kuramoto model on networks of varying topologies. The Kuramoto model is applied on the (A) random network, (B) scale-free network, (C) brain network of Gong et al., and (D) brain network of Hagmann et al., each with 78 nodes. For each network, the degree distribution and the mean directed phase lag index (dPLI) for each node averaged with respect to all other nodes are presented. Nodes are indexed in decreasing order of their degree; the degree distribution graph is red if the node degree is less than the average degree of the network, green if it is more than the average, and blue if it is higher than one standard deviation from the average. Mean dPLI values of each node are shown in color (blue/red representing lower/higher dPLI value) as their coupling strength changes from 0 to 5. A clear pattern is found in all networks, namely, the higher degree nodes have lower dPLI values and vice-versa. For Gong et al.'s network, delay time between two nodes was given proportional to distances between them with propagation speed of 6 m/s. For random, scale-free, and Hagmann et al.'s network the delay was assigned a value of 10ms. All simulations were performed 1000 times and averaged, and new random and scale-free network were generated with each simulation performed.
(EPS)

S4 Fig Stuart-Landau model on networks of varying topologies. The Stuart-Landau model was applied to a (A) random network, (B) scale-free network, (C) brain network of Gong et al., each with 78 nodes, and (D) brain network of Hagmann et al. with 66 nodes. The amplitudes and the mean directed phase lag index (dPLI) values for each node with respect to all other nodes are presented. Nodes of each network are indexed in decreasing order of their degree. Amplitudes and mean dPLI values of each node are shown in color (blue/red representing lower/higher amplitude and dPLI value) as their coupling strength changes from 0 to 5. A clear pattern is found in all networks, namely, the higher degree nodes have higher amplitudes and lower dPLI values (and vice-versa). For Gong et al.’s network, delay time between two nodes was given proportional to distances between them with propagation speed of 6 m/s. For random, scale-free, and Hagmann et al.’s network, the delay was assigned a value of 10ms. All simulations were performed 1000 times and averaged, and a new random and scale-free network was generated with each simulation performed.
(EPS)
