# Supplementary material for: General Relationship of Global Topology, Local Dynamics, and Directionality in Large-Scale Brain Networks
Source: PLoS Comput Biol. 2015 Apr 14;11(4):e1004225. doi: 10.1371/journal.pcbi.1004225 (PMC4397097; doi:10.1371/journal.pcbi.1004225)
Supplement: S1 Text — (PDF) [file pcbi.1004225.s001.pdf]

General Relationship of Global Topology, Local Dynamics, and Directionality in Large-Scale Brain Networks

Supporting Text

Contents

1 Relationship between complex models and Stuart-Landau/Kuramoto model 2

2 Directed phase lag index of Kuramoto model on complex networks 7

Kuramoto model on complex networks 8

Directed phase lag index of the Kuramoto model on complex networks . 13

3 Directed phase lag index of Stuart-Landau model on complex networks 15

Stuart-Landau model on complex networks 15

Directed phase lag index of Stuart-Landau model on complex networks 25

4 Comparison between different measures for the Stuart-Landau model 27

Here we report the main results and details of the mathematical analysis summarized in the main manuscript.

In section 1, we outline the relationship between more complex models and the simpler Stuart-Landau and Kuramoto models, demonstrating that the simple models used in the main manuscript are general lowest-order approximations of the more detailed neural mass models. In support of this, we show results of the derivation from the popular and complex Wilson-Cowan model as an example. In section 2, we describe the behavior of the Kuramoto model on complex networks. We also derive results of the directed phase lag index (dPLI) for the model on the networks. In section 3, we describe the Stuart-Landau model on complex networks, and derive the dPLI results for the model on the networks. In section 4, we also show with the simulation that the result from the dPLI and other measures such as Granger causality (GC) and symbolic transfer entropy (STE) qualitatively matches each other, suggesting that our results are independent from the choice of measures.

The mathematical analysis shown here provides a basis for the thesis of the main manuscript: *nodes with higher degrees are information sinks and lag in phase, whereas nodes with lower degrees are information sources and lead in phase*.

1 Relationship between complex models and Stuart-Landau/Kuramoto model

In this section we describe the relationship between more complex neural mass model, and the simpler Stuart-Landau and Kuramoto model. We also summarize the derivation from the popular and more detailed Wilson-Cowan model as an example.

Our investigation focuses on networks of coupled oscillators:

$$\dot{\mathbf{x}}_j = \mathbf{f}_j(\mathbf{x}_j) + \epsilon_j \mathbf{g}_j(x_1, ..., x_N, \epsilon, t), \qquad j = 1, 2, ..., N, \tag{S1}$$

where N is the number of oscillators, $\mathbf{x}_j \in \mathbb{R}^n$ is the state of the jth oscillator, therefore making $\mathbf{x} = (\mathbf{x}_1, \mathbf{x}_2, ..., \mathbf{x}_N)$ the vector describing the state of all oscillators of the network. $\mathbf{f}_j$ describes the intrinsic dynamics of the $\mathbf{x}_j$, and $\mathbf{g}_j$ describes the interaction of $\mathbf{x}_j$ with other oscillators. ϵ_j is the coupling strength for $\mathbf{x}_j$.

If the following condition is satisfied between two oscillators $\mathbf{x}_j$ and $\mathbf{x}_k$ they are called *frequency locked*,

$$n\omega_j = m\omega_k, \tag{S2}$$

where n and m are relatively prime nonnegative integers (they do not have a common divisor other than 1), and ω is the frequency of each oscillator. If $n : m$ is 1 : 1, then the oscillators are called *entrained*.

Suppose the oscillators are frequency locked. If they further satisfy the following condition,

$$|n\theta_j - m\theta_k| = constant, \tag{S3}$$

where θ is the phase of each oscillator, they are called *phase locked*. Note that frequency locking does not always imply phase locking; frequency locking without phase locking is called *phase trapping*.

If oscillators are both entrained and phase locked, they are then called *synchronous*. The quantity $\theta_{jk} = \theta_j - \theta_k$ is defined as *phase difference*, and when the phase difference between oscillators is zero, they are said to be *synchronized in-phase*. If not only their phases but also their amplitudes are also synchronized, they are *completely synchronized* (Amplitude synchronization means that their amplitudes are the same after transient period). When the phase difference is π they are called *synchronized anti-phase*, and if other than 0 or π, they are called *synchronized out-of-phase* [1, 2, 3].

If an entire network of oscillators satisfies such conditions, then the network can be said to be frequency locked, entrained, phase locked, synchronized, completely synchronized, etc.

Depending on its function $\mathbf{f}_j$, each oscillator in eq. (S1) can undergo various bifurcations as the parameters in their function change. One commonly observed bifurcation is termed the Hopf bifurcation. Near the Hopf bifurcation point, function $\mathbf{f}_j$ of each oscillator can be approximated well as the following pair of equations [4, 5]:

$$\begin{aligned} \dot{x}_j &= \lambda_j x_j - \omega - j y_j \mp (\sigma_j x_j - \gamma_j y_j)(x_j^2 + y_j^2), \\ \dot{y}_j &= \lambda_j y_j + \omega_j x_j \mp (\sigma_j y_j + \gamma_j x_j)(x_j^2 + y_j^2), \end{aligned} \tag{S4}$$

or, in complex coordinates, taking $z = re^{i\theta} = x + iy$,

$$\dot{z}_j = \{\lambda_j + i\omega_j \mp (\sigma_j + i\gamma_j)|z_j|^2\}z. \tag{S5}$$

In polar coordinates, it can be written as,

$$\begin{aligned} \dot{r}_j &= \{\lambda_j \mp \sigma_j|z_j|^2\}r_j, \\ \dot{\theta}_j &= \omega_j \mp \gamma_j|z_j|^2. \end{aligned} \tag{S6}$$

Here, λ, ω, σ, and γ are nonnegative coefficients. These equations are the normal form for the Hopf bifurcation and are called *Stuart-Landau equation*. $\mp$ determines whether the bifurcation be supercritical or subcritical: $-$ for superciritical Hopf bifurcation, and $+$ for subcritical. Dynamics of the equations for $\gamma = 0$ and $\gamma \neq 0$ are topologically equivalent, so the value of γ is often irrelevant.

With coupling terms, we rewrite the above equations as:

$$\begin{aligned} \dot{x}_j &= \lambda_j x_j - \omega_j y_j \mp (\sigma_j x_j - \gamma_j y_j)(x_j^2 + y_j^2) + \sum_{k=1}^{N} K_{jk} x_k, \\ \dot{y}_j &= \lambda_j y_j + \omega_j x_j \mp (\sigma_j y_j + \gamma_j x_j)(x_j^2 + y_j^2) + \sum_{k=1}^{N} K_{jk} y_k, \end{aligned} \tag{S7}$$

in complex coordinates,

$$\dot{z}_j = \{\lambda_j + i\omega_j \mp (\sigma_j + i\gamma_j)|z_j|^2\}z + \sum_{k=1}^{N} K_{jk} z_k, \tag{S8}$$

in polar coordinates,

$$\begin{aligned} \dot{r}_j &= \{\lambda_j \mp \sigma_j|z_j|^2\}r_j + \sum_{k=1}^{N} K_{ij} r_k \cos(\theta_k - \theta_j), \\ \dot{\theta}_j &= \omega_j \mp \gamma_j|z_j|^2 + \sum_{k=1}^{N} K_{ij} \frac{r_j}{r_k} \sin(\theta_k - \theta_j), \end{aligned} \tag{S9}$$

describing the state of node j. Here, K_{jk} is the coupling strength from k to j. We refer to this system of equations as the *Stuart-Landau model*. More details on the Hopf bifurcation and the Stuart-Landau model will be discussed in section 3.

Reduction of eq. (S1) to simpler equations can be made even further. If $\epsilon_j \ll 1$, $\mathbf{f}_j$ in eq. (S1) can be reduced to a phase equation describing the state of node j only by its phase [1]:

$$\dot{\theta}_j = \omega_j. \tag{S10}$$

With the coupling term, we have the following equation:

$$\dot{\theta}_j = \omega_j + \sum_{k=1}^{N} K_{ij} \sin(\theta_k - \theta_j). \tag{S11}$$

This is the generalization of the well-known *Kuramoto model* [6, 7]. Although this equation is a generalization and not exactly the same as the original form of the Kuramoto model (in which K_{ij} is equal for all is and js), for brevity, we refer to this equation as Kuramoto model hereafter. Notice that this equation can be derived simply from the phase equation of the Stuart-Landau model, eq. (S9), by setting all the amplitudes of the oscillators to be equal and static.

It is known that the long-term behavior of any coupled oscillatory systems, not only the systems with the Hopf bifurcation, can be approximated by coupled phase oscillators of the form,

$$\dot{\theta}_j = \omega_j + \sum_{k=1}^{N} K_{jk} H(\theta_k - \theta_j), \tag{S12}$$

as long as the coupling is not too strong and the subsystems are nearly identical [1, 6, 7, 12]. We arrive at the Kuramoto model, eq. (S11), by setting $H(\theta_k - \theta_j) = \sin(\theta_k - \theta_j)$. The Kuramoto model is the first-order approximation to the general form of coupled phase oscillators eq. (S12). In this sense, the Kuramoto model is the *canonical model* of coupled oscillators.

The Wilson-Cowan model is one of the most popular neural mass models with two equations describing the state of excitatory and inhibitory cell populations [8, 9]. We state the results of the derivation of the Stuart-Landau model and Kuramoto model from the Wilson-Cowan model as an example of the described approximation scheme. The results adopted here come from references [10, 11] and [12].

We define the Wilson-Cowan model as a network of oscillators with dynamics at node j described as:

$$\dot{E}_j = -E_j + S[a_E(c_{EE}E_j - c_{IE}I_j - \rho_E + P_j + \eta\sum_{k=1}^{N} A_{jk}E_k)],$$
$$\dot{I}_j = -I_j + S[a_I(c_{EI}E_j - c_{II}I_j - \rho_I + Q_j)], \tag{S13}$$

here, a_E, a_i, c_{EE}, c_{EI}, c_{IE}, c_{II}, ρ_E, ρ_I, P_j, and Q_j are the positive coefficients, η is the coupling strength, and A_{jk} is the coupling strength between j and k. S is a sigmoid function, usually given as $S[x] = (1 - e^{-x})^{-1}$. Here, the interaction term is only added to the equation describing the excitatory population, E_j, because non-local neural mass connections are usually made by the excitatory cells.

If the parameters are given suitably eq. (S13) will yield a stable limit cycle, i.e., a stable oscillatory trajectory. The strategy is to expand the sigmoid function S of eq. (S13) in this oscillatory regime, removing the higher-order terms. Then, the resulting approximated equations will be averaged over one cycle. This is made possible by the assumption that the amplitude and the phase of the oscillators will change slowly compared to the oscillators' frequency. For the averaging, time-dependent amplitude and phase are fixed, and the system is integrated over one period. Subsequently, amplitude and phase are again considered to be time-dependent: this procedure is called the method of averaging. The resulting equations reproduces the normal form of Hopf bifurcation. The equations can be further simplified into phase equations by assuming that the amplitudes do not change (or change very little compared to the phases).

After *1*) expanding eq. (S13) around the unstable fixed-point $(E_j^{(0)}, I_j^{(0)})$ within the stable limit cycle, with respect to the sigmoid function S[x], *2*) abandoning higher order terms of the expansion S[x], and *3*) averaging over a cycle $t = [0, 2\pi/\Omega)$, where Ω is the mean frequency of the whole oscillators, we get:

$$\dot{r}_j \approx \lambda_j r_j + \sigma_j r_j^3 + \sum_{k=1}^{N} a_E S'[\chi_{E,j}^{(0)}] r_k A_{jk} \cos(\theta_k - \theta_j),$$
$$\dot{\theta}_j \approx \omega_j + \frac{1}{2}\sum_{k=1}^{N} a_E S'[\chi_{E,j}^{(0)}] \frac{r_k}{r_j} A_{jk} \sin(\theta_k - \theta_j), \tag{S14}$$

where

$$\omega_j \approx -\Omega + \frac{1}{2}(a_E c_{IE} S'[\chi_{E,j}^{(0)}] + a_I c_{EI} S'[\chi_{I,j}^{(0)}]),$$
$$\lambda_j = \frac{1}{2}(a_E c_{IE} S'[\chi_{E,j}^{(0)}] - a_I c_{EI} S'[\chi_{I,j}^{(0)}] - 2), \tag{S15}$$
$$\sigma_j = \frac{1}{16}(a_E^3 c_{EE}(c_{EE}^2 + c_{IE}^2) S'''[\chi_{E,j}^{(0)}] - a_I^3 c_{II}(c_{II}^2 + c_{EI}^2) S'''[\chi_{I,j}^{(0)}] - 2).$$

$S'[x]$ and $S'''[x]$ are first and third derivatives of S at x. We used the abbreviation:

$$\chi_{E,j}^{(0)} = a_E(c_{EE} E_j^{(0)} - c_{IE} I_j^{(0)} - \rho_E + P_j + \eta \sum_{k=1}^{N} A_{jk} E_k^{(0)}),$$
$$\chi_{I,j}^{(0)} = a_I(c_{EI} E_j^{(0)} - c_{II} I_j^{(0)} - \rho_I + Q_j). \tag{S16}$$

This is exactly the normal form of Hopf bifurcation, i.e., Stuart-Landau model, and if $\sigma_j < 0$ the oscillator will undergo the supercritical Hopf bifurcation.

If we assume that all amplitudes r_j are small (and equal to each other), such that we can discard all the terms with r_j or higher-order (and to set $r_j/r_k = 1$), we finally arrive at the Kuramoto model:

$$\dot{\theta}_j = \omega_j + \frac{1}{2}\sum_{k=1}^{N} a_E S'[\chi_{E,j}^{(0)}] A_{jk} \sin(\theta_k - \theta_j). \tag{S17}$$

In this context, i.e., Stuart-Landau and Kuramoto models as the approximations for more complex neural mass models, we use these models to explain the directionality of the information flow across networks.

2 Directed phase lag index of Kuramoto model on complex networks

In this section we apply the Kuramoto model to complex networks with an broad degree distribution such as random and scale-free networks [13], and calculate directed phase lag index (dPLI) of the model on the network. The model used here is similar to that of reference [14], and the method used to solve the model in that reference can be adopted here to solve our model. The analysis of reference [14] includes a similar line of arguments as reference [15].

Kuramoto model on complex networks

As shown in section 1 and in the references [1, 6, 7, 12], systems of coupled oscillators can be reduced to the following general form of phase-only equations as the lowest order approximation:

$$\dot{\theta}_j = \omega_j + \sum_{k=1}^{N} K_{jk} H(\theta_k - \theta_j), \qquad j = 1, 2, ..., N, \tag{S18}$$

where $\dot{\theta}_j(t)$ is the phase of oscillator j at time t, ω_j is the natural frequency of the oscillator j, and N is the total number of oscillators. K_{jk} is the coupling strength from oscillator k to oscillator j. $H(\theta)$ is the coupling function. This is the most general form of the phase model for coupled oscillators. In our model, the coupling function $H(\theta)$ is sin(θ).

Our model also requires finite transmission delays τ between different oscillators, emulating the delay of signal propagation between two neural mass populations:

$$\dot{\theta}_j(t) = \omega_j + \sum_{k=1}^{N} K_{jk} \sin(\theta_k(t-\tau) - \theta_j(t)), \qquad j = 1, 2, ..., N. \tag{S19}$$

This is the equation we use in our simulation as the neural mass model for brain networks. The natural frequencies in our simulation are given as a Gaussian distribution with a mean at 10 Hz and standard deviation 1, making ω_j about $10{\cdot}2\pi$ rad/s. Time delay is varied between $2 \sim 50$ ms in our simulation, but the value of delay does not bring about qualitative differences of the outcome, as long as it is less than a quarter of the time of one cycle for the natural frequency (in this case, given the frequency of 10 Hz, the time for one cycle is 100 ms).

In order to analyze this model, we follow the mean-field technique used by Ko et al. [14]. As an approximation of the model in Eq. (S19), we write

$$\dot{\theta}_j(t) = \omega_j + K_j \sum_{k=1}^{N} \sin(\theta_k(t-\tau) - \theta_j(t)), \qquad j = 1, 2, ..., N, \tag{S20}$$

where K_j corresponds to the average coupling strength to oscillator j. Through this mean-field approximation, the coupling inhomogeneity is incorporated in K_j,

and the model becomes easier to analyze. We analyze this model to study coupling inhomogeneity, and relate the simulation results to networks with inhomogeneous degree distribution (e.g., random network, scale-free network and brain network).

Reference [16] states that if the time delays between the oscillators are similar or smaller in their order of magnitude compared to their oscillatory period, there will be no explicit time delay term but rather represented as a phase delay term β in the coupling function: $H(\theta - \beta)$. Normalization factor $1/N$ will be added to the coupling strength for the ease of analysis. Taking into account of these changes, we finally arrive at our equation of analysis:

$$\dot{\theta}_j = \omega_j + \frac{K_j}{N} \sum_{k=1}^{N} \sin(\theta_k - \theta_j - \beta), \qquad j = 1, 2, ..., N, \tag{S21}$$

at time t.

Our model Eq. (S21) is a simplified version of Ko et al.'s from the reference [14]. Analytic techniques and results from the reference can also be applied to our model. We summarize the behaviors of the model that are necessary in explaining the phase-lead/lag relationship between oscillators of the model. Compared to the given natural frequencies ω_j, the nonzero phase delay β is going to be small enough to assume that $\beta \in (0, \pi/2)$. With this condition, we obtain a so-called *partially locked state* as the possible solution of Eq. (S21) [14]. In terms of our original model in Eq. (S19), if the coupling strength K between each node is increased from 0, the system as a whole will change from an incoherent state to a partially locked state before reaching phase locked state. What follows is a more detailed description of the nontrivial state, i.e., the partially locked state.

In a partially locked state, the oscillators are divided into a phase locked group oscillating together, and a drifting group with different frequencies and phases than the locked group. This partially locked state can be analyzed using a self-consistency argument [10, 11, 14]. We first introduce a parameter R:

$$Re^{i\Theta} = \frac{1}{N} \sum_{k=1}^{N} e^{i\theta_k}. \tag{S22}$$

R is an order parameter having values between 0 and 1; 0 indicates uniform incoherence, and 1 indicates in-phase synchrony.

Let Ω denote the frequency of the population oscillation of Eq. (S22) after the system approaches a stationary state and let $\phi_j = \theta_j - \Omega t$ represent the phase of

oscillator j relative to the average oscillation. The Eq. (S21) can then be rewritten using the order parameter defined in Eq. (S22) as follows:

$$\dot{\phi}_j = \omega_j - \Omega + K_j R \sin(\Phi - \phi_j - \beta), \qquad j = 1, 2, ..., N, \tag{S23}$$

where $\Phi = \Theta - \Omega t$. When the system reaches a stationary state, R and Φ do not depend on time.

The condition for the oscillators to be phase locked is $\dot{\phi}_j = 0$.Then the amplitude of the coupling terms must be larger than the inherent terms:

$$K_j R > |\omega_j - \Omega|. \tag{S24}$$

From the simulation result, we found that $\omega_j - \Omega > 0$, which means that the average frequency of the oscillators will be lower than the initially given frequencies: the oscillators slow down as they synchronize with each other. We can also show this analytically.

The oscillators satisfying the above condition in Eq. (S24) will asymptotically approach a stable fixed point ${\phi_j}^*$ obtained from the following equation:

$$\omega_j - \Omega = K_j R \sin({\phi_j}^* - \Phi + \beta). \tag{S25}$$

Three cases are possible:

$$\begin{aligned} &case\ i) \quad \omega_j - \Omega < 0 \quad implying \quad \pi < {\phi_j}^* - \Phi + \beta < 2\pi, \\ &case\ ii) \quad \omega_j - \Omega = 0 \quad implying \quad {\phi_j}^* - \Phi + \beta = 0 \ or\ \pi, \\ &case\ iii) \quad \omega_j - \Omega > 0 \quad implying \quad 0 < {\phi_j}^* - \Phi + \beta < \pi. \end{aligned} \tag{S26}$$

Also, the stability condition for the fixed point is

$$\cos({\phi_j}^* - \Phi + \beta) > 0, \tag{S27}$$

leading to,

$${\phi_j}^* - \Phi + \beta \in (-\pi/2, \pi/2). \tag{S28}$$

Applying Eq. (S28) reduces Eq. (S26) into:

$$\begin{aligned} &case\ i) \quad 3\pi/2 < {\phi_j}^* - \Phi + \beta < 2\pi, \\ &case\ ii) \quad {\phi_j}^* - \Phi + \beta = 0, \\ &case\ iii) \quad 0 < {\phi_j}^* - \Phi + \beta < \pi/2, \end{aligned} \tag{S29}$$

or,

$$\begin{aligned} case\ i) \quad & 3\pi/2 - \beta < {\phi_j}^* - \Phi < 2\pi - \beta, \\ case\ ii) \quad & {\phi_j}^* - \Phi = -\beta, \\ case\ iii) \quad & 0 - \beta < {\phi_j}^* - \Phi < \pi/2 - \beta. \end{aligned} \tag{S30}$$

Since Φ represents the phase of the average oscillation of all oscillators, ${\phi_j}^* - \Phi$ must be able to have both negative values and positive values. With $\beta \in (0, \pi/2)$, the only case yielding such possibility is $case\ iii$. Therefore, avoiding contradiction, $\omega_j - \Omega > 0$.

Applying this result to the above condition Eq. (S24), we can state the condition for node j to phase lock as:

$$K_j R > \omega_j - \Omega. \tag{S31}$$

We also find

$${\phi_j}^* - \Phi + \beta \in (0, \pi/2). \tag{S32}$$

We will use these findings in the calculation of the dPLI for the model.

The oscillators satisfying the above condition Eq. (S31) are phase locked at frequency Ω in the original frame. The oscillators with $K_j R < \omega_j - \Omega_j$ will not be able to lock and will drift monotonically.

If we assume that the initial frequencies for each node j are given identically ($\omega_j = \omega$ for $j = 1, 2, ..., N$), we can further write the following expression as the condition for the oscillators to phase lock:

$$K_j > \frac{\omega - \Omega}{R} \equiv K_l. \tag{S33}$$

From this condition, the oscillators that phase lock are the ones with their $K_j > K_l$,

$$D_l = \left\{ K_j : K_l < K_j \right\}, \tag{S34}$$

and the oscillators that drift are the ones with their $K_j < K_l$,

$$D_d = \left\{ K_j : K_j < K_l \right\}. \tag{S35}$$

As noted, these results are applicable to the Kuramoto model on networks [14].

$$\dot{\theta}_j = \omega_j + S\sum_{k=1}^{N} A_{jk}\sin(\theta_k - \theta_j - \beta), \qquad j = 1, 2, ..., N, \tag{S36}$$

where S is the coupling strength, and A is the adjacency matrix describing the coupling topology of the network. We will denote the incoming degree (number of connections from other nodes) of node j by k_j, and let A_{jk} be either 1 (if there exits a connection from k to j) or 0 (if there is no connection between j and k). If the oscillator connections are random, we can use the following approximation [14, 17]:

$$S\sum_{k=1}^{N} A_{jk}H(\theta_k - \theta_j) \approx \frac{Sk_j}{N}\sum_{k=1}^{N} H(\theta_k - \theta_j). \tag{S37}$$

With such relation, Eq. (S36) is approximately equivalent to the following equation:

$$\dot{\theta}_j = \omega_j + \frac{Sk_j}{N}\sum_{k=1}^{N} \sin(\theta_k - \theta_j - \beta), \qquad j = 1, 2, ..., N, \tag{S38}$$

which is equivalent to Eq. (S21) with $K_j = Sk_j$.

Whether a specific given network indeed satisfies the above approximation can be confirmed by comparison with simulation results. However, provided there exist sufficient connections between different "communities" of the network, and because of its *small-world* property that allows short-cuts across the network, it is known that the above mean-field approximation approach holds well for sufficiently strong coupling strength S such that clusters of entrained oscillators are being formed [17].

The condition for a node j to phase lock is:

$$Sk_jR > \omega_j - \Omega, \tag{S39}$$

and when $\omega_j = \omega$ for $j = 1, 2, ..., N$, Eq. (S33) can be restated:

$$Sk_j > \frac{\omega - \Omega}{R} \equiv Sk_l, \tag{S40}$$

k_l being the critical degree. Nodes with degree larger than k_l will phase lock, and less than k_l will drift. As the coupling strength S gets larger, the system will eventually reach the fully locked state.

Directed phase lag index of the Kuramoto model on complex networks

We use directed phase lag index (dPLI) as the measure of direction of information flow. Building on phase lag index (PLI) from reference [18], dPLI was first defined in reference [19].

If two oscillators are phase locked, the condition Eq. (S3) is satisfied. Moreover if their frequencies are 1:1, Eq. (S3) can be rewritten:

$$|\theta_{jk}| = |\theta_j - \theta_k| = constant, \tag{S41}$$

where θ_{jk} is the phase difference between node j and k. Given a time series, phase differences at each time can be computed: $\theta_{jk}(t)$ for $t = 1, 2, ..., T$. PLI between two oscillators j and k is defined as the absolute value of the time average of the sign of $\theta_{jk}(t)$:

$$PLI_{jk} = |\langle sign\{\theta_{jk}(t)\}\rangle|. \tag{S42}$$

The PLI ranges between 0 and 1: a PLI of zero indicates no coupling or coupling with a phase difference centered around 0 mod π, and a PLI of 1 indicates perfect phase locking at a difference other than 0 mod π. The stronger this nonzero phase locking is, the closer to 1 PLI will be. The PLI does not reflect the magnitude of the phase difference, or the direction.

dPLI was defined to indicate the direction of information flow. This measure reflects which of the two signals is leading and which is lagging in phase. Here we define dPLI as the PLI without taking the absolute value of:

$$dPLI_{jk} = \langle sign\{\theta_{jk}(t)\}\rangle, \tag{S43}$$

or, equivalently, as the following:

$$dPLI_{jk} = \frac{1}{T}\sum_{t=1}^{T} \tilde{H}(\theta_{jk}(t)), \tag{S44}$$

here, $\tilde{H}(x) \equiv 2H(x) - 1$, where $H(x)$ is the Heaviside step function yielding values either 0 (if $x < 0$) or 1 (if $x \geq 0$). $\tilde{H}_{jk}(x)$ will yield values either -1

(if j is phase lagging compared to k) or 1 (if j is phase leading compared to k). Therefore, $dPLI_{jk}$ will yield 1 if j is always phase leading compared to k, and -1 if j is always phase lagging. If a phase-lead/lag relationship between two arbitrary nodes can be analyzed, we can automatically predict whether the dPLI values will be positive or negative between those nodes. In this sense, finding dPLI and phase-lead/lag relationship is equivalent.

We now derive phase-lead/lag relationships among oscillators of the network. From the previous subsection, we know oscillators that are phase locked to each other satisfy Eq. (S25),

$$\omega_j - \Omega = K_j R \sin({\phi_j}^* - \Phi + \beta). \tag{S45}$$

We also know that ${\phi_j}^* - \Phi + \beta \in (0, \pi/2)$. Given two oscillators ϑ and φ, if we assume their given frequencies are equal to each other, $\omega_\vartheta = \omega_\varphi$, and their average coupling strengths K_ϑ and K_φ have the relation $K_\vartheta < K_\varphi$, we can write:

$$K_\vartheta R \sin({\phi_\vartheta}^* - \Phi + \beta) = K_\varphi R \sin({\phi_\varphi}^* - \Phi + \beta). \tag{S46}$$

From ${\phi_j}^* - \Phi + \beta \in (0, \pi/2)$, we know that sin terms on both side of the equation is positive and monotonically increasing. Therefore, for phase locked oscillators, if $K_\vartheta < K_\varphi$, then ${\phi_\vartheta}^* - \Phi + \beta > {\phi_\varphi}^* - \Phi + \beta$, which leads to the following relation: ${\phi_\vartheta}^* > {\phi_\varphi}^*$.

To summarize, for phase locked oscillators,

$$if \quad K_\vartheta < K_\varphi \quad then \quad {\phi_\vartheta}^* > {\phi_\varphi}^*. \tag{S47}$$

We can interpret these results for inhomogeneous networks via step of Eq. (S36) to Eq. (S38). From $K_j = Sk_j$, the higher the degree of node j,k_j, is, the larger the value of K_j becomes. Therefore, if the degree of node j is higher, it will phase lag, and lower degree nodes will phase lead. If the coupling strength S increases, more and more oscillators will phase lock to each other. Therefore, the larger the coupling strength S is, the more apparent the phase-lead/lag relationship will be.

Our simulation results are shown in S3 Figure, and confirm our analytic results. In the derivations, we assumed constant time delay τ and therefore constant phase delay β between oscillators. We also assumed that the given natural frequencies of the oscillators are all equal to each other: $\omega_j = \omega$. In the simulations however, distance-dependent time delay as well as constant time delay were applied in the case of Gong's anatomic human brain network. The result is similar, always showing negative correlation between the node degree and dPLI. The simulations show

that the analytical results hold well for distance-dependent time delays as long as the delays are smaller than one quarter of one oscillating cycle, as is the case with the constant time delays. Additionally, the natural frequencies of the oscillators were given as a Gaussian distribution with mean at 10 Hz and standard deviation 1 (making ω_j around 10·2π rad/s). The result were again similar in the case where all natural frequencies were equal ($\omega_j = \omega$). The variations in the time delay τ and ω_j will act as perturbations to each oscillators while maintaining the overall tendency of the negative correlation. In addition to above perturbations, we also added a Gaussian white noise $\xi_j(t)$ of vanishing mean and standard deviation of 2 to each oscillator's equation to test the robustness of our results against perturbations. Against all these perturbations, as shown in S3 Figure, the main finding of the analysis was still maintained: higher degree nodes phase-lag, whereas lower degree nodes phase-lead.

3 Directed phase lag index of Stuart-Landau model on complex networks

In this section we describe the Stuart-Landau model on scale-free networks [20, 21], and derive dPLI for the model on the network. The method used to analyze the Kuramoto model can be applied here as well.

Stuart-Landau model on complex networks

As shown in section 1, the Stuart-Landau model can be derived as the normal form for the Hopf bifurcation. The Wilson-Cowan model was given as an example: the model was expanded to the range of solutions yielding a stable limit cycle, and the resulting low-order approximation was equivalent to the Stuart-Landau model.

The Stuart-Landau model is written as:

$$\dot{z}_j = \{\lambda_j + i\omega_j - (\sigma_j + i\gamma_j)|z_j|^2\}z_j + \sum_{k=1}^{N} K_{jk} z_k, \qquad j = 1, 2, ..., N, \tag{S48}$$

where complex variable $\dot{z}_j(t)$ describes the state of jth oscillator.

For the moment, let us first consider the model without the coupling term, that is, the inherent part of the model only:

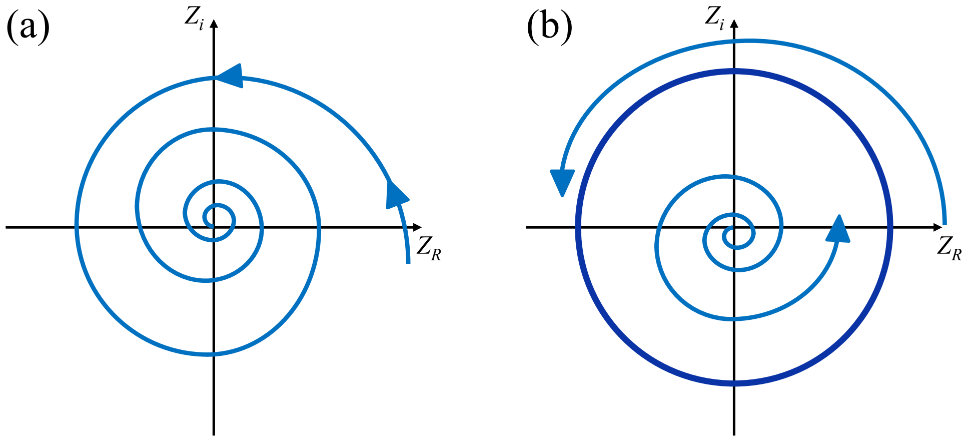

Illustration 1: Eq. (S49) with $\sigma_j > 0$ and $\omega > 0$. $\sigma_j > 0$ makes it a case of supercritical bifurcation, chainging from stable focus (a) to stable limit cycle with an unstable focus at the center. $\omega > 0$ makes the trajectory rotate counter-clockwise. If $\omega < 0$ the trajectory rotates clockwise. (a) When $\lambda_j < 0$ there is a stable focus at the center. (b) When $\lambda_j > 0$ stable focus changes to unstable focus, and a stable limit cycle appears.

$$\dot{z}_j = \{\lambda_j + i\omega_j - (\sigma_j + i\gamma_j)|z_j|^2\}z_j. \tag{S49}$$

In this equation, λ_j is a parameter controlling how fast the trajectory decays onto the attractor, ω_j is the natural frequency of each oscillator, and γ_j is the coupling term between the amplitude and phase of the oscillator. The sign of σ_j decides whether the Hopf bifurcation is supercritical ($\sigma_j > 0$) or subcritical ($\sigma_j < 0$).

Here, we assume that $\lambda, \omega, \sigma,$ and γ are all nonnegative. Then the equation yields a stable limit cycle from the supercritical Hopf bifurcation [20, 21]. A stable limit cycle appears via a superciritical Hopf bifurcation when $\lambda_j > 0$, where as when $\lambda_j < 0$ there is only a stable focus at the center (the point of bifurcation is $\lambda_j = 0$). Illustration 1 shows the behaviour of the equation Eq. (S49) in the case of $\sigma_j > 0$. The dynamics of the equations for $\gamma = 0$ and $\gamma \neq 0$ are topologically equivalent, so the value of γ is often irrelevant. For the ease of analysis, we set $\gamma = 0$ and $\sigma = 1$. Also, we again add time-delay τ between nodes.

Returning back to the model with the coupling term, for each node j, the dynamics will be:

$$\dot{z}_j(t) = \{\lambda_j + i\omega_j - |z_j(t)|^2\} z_j(t) + \sum_{k=1}^{N} K_{jk} z_k(t - \tau). \tag{S50}$$

This is the form of the model we use in our simulation. We set $\lambda_j = 2$ for all $j = 1, 2, ..., N$, and again give a Gaussian distribution with a mean at 10 Hz and a standard deviation 1 for natural frequencies, making ω_j about $10 \cdot 2\pi$ rad/s. The time delay is varied between $2 \sim 50$ ms, but again does not result in qualitative differences as long as it is smaller than a quarter the time of one cycle for the natural frequency (25 ms).

We again use the mean-field approximation technique of reference [14] as used in the section 2 to analyze this model. As an approximation of the model Eq. (S50), we write:

$$\dot{z}_j(t) = \{\lambda_j + i\omega_j - |z_j(t)|^2\} z_j(t) + K_j \sum_{k=1}^{N} z_k(t - \tau), \tag{S51}$$

where K_j corresponds to the average coupling strength to oscillator j. Again, the coupling inhomogeneity is incorporated in K_j. We analyze this model to study coupling inhomogeneity and relate the simulation results from networks with inhomogeneous degree distribution (e.g., random network, scale-free network and brain network).

As in section 2, we use the result from reference [16]: if the time delays between the oscillators are similar or smaller compared to their oscillatory period, time delay can be represented generically as a phase delay term β without an explicit time delay term. Normalization factor $1/N$ will be added to the coupling strength for the ease of analysis. We arrive at the following equation for each node j:

$$\dot{z}_j = \{\lambda_j + i\omega_j - |z_j|^2\} z + \frac{K_j}{N} \sum_{k=1}^{N} z_k(t) e^{-i\beta}, \tag{S52}$$

at time t. Eq. (S52) can be separated into two variables:

$$\dot{r}_j = \{\lambda_j - |z_j|^2\} r_j + \frac{K_j}{N} \sum_{k=1}^{N} r_k \cos(\theta_k - \theta_j - \beta), \tag{S53}$$

$$\dot{\theta}_j = \omega_j + \frac{K_j}{N}\sum_{k=1}^{N}\frac{r_k}{r_j}\sin(\theta_k - \theta_j - \beta). \tag{S54}$$

These are the equations to be used in the analysis. $r_j(t)$ is the amplitude of node j, and $\theta_j(t)$ is the phase of node j at time t.

We define a new parameter for the Stuart-Landau model for our analysis:

$$\tilde{R}e^{i\Theta} = \frac{1}{N}\sum_{j=1}^{N} r_j e^{i\theta_j}. \tag{S55}$$

$\tilde{R}$ is a generalization of R defined in Eq. (S22): $Re^{i\Theta} = \frac{1}{N}\sum_{j=1}^{N} e^{i\theta_j}$. This new parameter $\tilde{R}e^{i\Theta}$ is a sum of all the z_js in the network, and $\tilde{R}$ can have values near 0 (when they are in uniform incoherence), to $\frac{1}{N}\sum_{j=1}^{N} r_j$, the mean of amplitude of the oscillators (when they are in-phase synchronized). When they are completely synchronized, their amplitudes are all equal to each other (like the phases are) and the value $\tilde{R}$ will equal their amplitudes, r_j.

Denoting Ω the frequency of the population oscillation of Eq. (S55) after the system approaches a stationary state and setting $\phi_j = \theta_j - \Omega t$ the phase of oscillator j relative to the average oscillation, the Eq. (S53) and (S54) can be written using the new order parameter Eq. (S55) as follows for each node j:

$$\dot{r}_j = \{\lambda_j - {r_j}^2\}r_j + K_j\tilde{R}\cos(\Phi - \phi_j - \beta), \tag{S56}$$

$$\dot{\phi}_j = \omega_j - \Omega + K_j\frac{\tilde{R}}{r_j}\sin(\Phi - \phi_j - \beta), \tag{S57}$$

where $\Phi = \Theta - \Omega t$. $\tilde{R}$ and Φ will not depend on time when the system reaches a stationary state. We can also write in one equation form,

$$\dot{z}_j = \{\lambda_j + i(\omega_j - \Omega) - |z_j|^2\}z + K_j\tilde{R}e^{i(\Phi-\beta)}. \tag{S58}$$

The result is in the form of a Stuart-Landau equation with a forcing term; such an equation has been studied in references [22, 23, 24]. Here we focus on the relations between the amplitude r_j, the strength K_j and the phase-lead/lag of node j compared to each other.

The phase equation Eq. (S57) is similar to the equation for the Kuramoto model Eq. (S23): $\dot{\phi}_j = \omega_j - \Omega + K_jR\sin(\Phi - \phi_j - \beta)$, with the difference being a factor $1/r_j$, and $\tilde{R}$ instead of R. The simulation shows similarity to the Kuramoto model such that $\omega_j - \Omega > 0$, as synchronized frequency of the oscillators is lower than

the initially given frequencies. However, we do not have to rely on the simulation. Similar analysis done for Eq. (S23) in the previous section can also be applied to Eq. (S57). When the system reaches a stationary state, $\tilde{R}$ and Φ do not depend on time. Repeating the analysis done from Eq. (S24) to Eq. (S30) for Eq. (S57), we again find $\omega_j - \Omega > 0$. Also, we find the condition for node j to phase lock:

$$K_j \frac{\tilde{R}}{r_j} > \omega_j - \Omega. \tag{S59}$$

We also find, again,

$${\phi_j}^* - \Phi + \beta \in (0, \pi/2). \tag{S60}$$

As the coupling strength gets larger, the synchronized frequency decreases. However, there exists a notable difference between the Stuart-Landau model and the Kuramoto model. For a sufficient coupling strength the population is divided into two groups in the Kuramoto model, a phase locked group and a drifting group, before they phase lock as one group for even larger coupling strength. For the Stuart-Landau model, the oscillators phase lock as one group more instantaneously. The reason for this difference will become apparent later.

If the system indeed reaches the stationary state so that the oscillator j of the model asymptotically reaches a stable value $(r_j^*,\ \phi_j^*)$, Eq. (S56) and (S57) can be written as:

$$-\{\lambda_j - r_j^{*2}\} r_j^* = K_j \tilde{R} \cos(\phi_j^* - \Phi + \beta), \tag{S61}$$

$$\omega_j - \Omega = K_j \frac{\tilde{R}}{r_j^*} \sin(\phi_j^* - \Phi + \beta). \tag{S62}$$

The solution $(r_j^*,\ \phi_j^*)$ to the equations is the stable fixed point we sought after. If we square both equations and add them together, we arrive at the following expression:

$$r_j^{*2}\{(\lambda_j - r_j^{*2})^2 + (\omega_j - \Omega)^2\} = (K_j \tilde{R})^2. \tag{S63}$$

This is a cubic equation for r_j^{*2}. Being a cubic equation with real coefficients, up to three real solutions are possible, and at least one real solution exists [22, 23]. The Tartaglia explicit formula for cubic equations are available to solve this equation, but is extremely involved. However we can utilize graphical methods to draw out useful information [24].

We first rearrange the equation with substitutions. With

$$\begin{cases} x \equiv r_j^{*2}, \\ A \equiv \lambda_j, \\ B \equiv \omega_j - \Omega, \end{cases} \tag{S64}$$

we set

$$g(x; A, B) \equiv x\{(A-x)^2 + B^2\}. \tag{S65}$$

The squared amplitude of the phase locked solution $x \equiv r_j^{*2}$ is obtained from the solutions to $g(x) = (K_j\tilde{R})^2$. We look for positive real solutions. Graphically, we look for intersections of the function $g(x)$ with a horizontal line drawn at height $(K_j\tilde{R})^2$ above the x-axis. There exists at least one intersection always. There are two possible cases for the intersections to occur as depicted in Illustration 2, depending on the parameters A and B. $g(x)$ may be monotonically increasing and there will exist one positive real solution (*case i*), or $g(x)$ may have local maximum and minimum at positive x values x_{max} and x_{min} before monotonically increase (*case ii*). In the *case ii*, if $g(x_{min}) < (K_j\tilde{R})^2 < g(x_{max})$ there will exist three positive real solutions. the proof of existance for such solutions for both cases are well documented in the reference [22].

We can calculate the condition for the *case ii*. If we take the derivative of $g(x)$,

$$\frac{\partial g(x; A, B)}{\partial x} = 3x^2 - 4Ax + (A^2 + B^2). \tag{S66}$$

The condition for the $g(x)$ to be *case ii* is, for the following equation to have two solutions which are non-imaginary:

$$\frac{\partial g(x; A, B)}{\partial x} = 0. \tag{S67}$$

The solution to Eq. (S67) is

$$x_{\pm} = \frac{2A}{3} \pm \frac{\sqrt{A^2 - 3B^2}}{3}, \tag{S68}$$

and for $x_{\pm}$ to be two different non-imaginary values,

$$A^2 > 3B^2, \tag{S69}$$

or,

$$\lambda_j{}^2 > 3(\omega_j - \Omega)^2. \tag{S70}$$

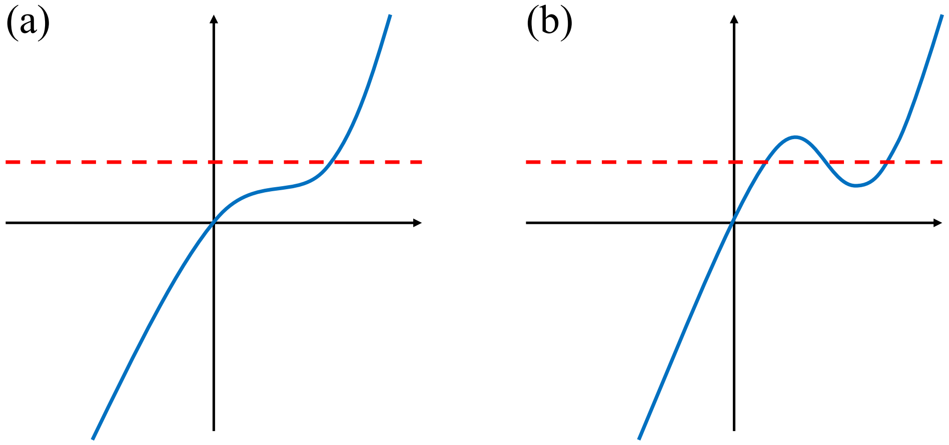

Illustration 2: Possible cases for $g(x) = (K_j\tilde{R})^2$. $g(x)$ is a blue curve, and $(K_j\tilde{R})^2$ is a red dashed-line. (a) $g(x)$ may monotonically increase, or (b) $g(x)$ may have local maximum and minimum at positive x values x_{max} and x_{min} before monotonically increase. If $g(x_{min}) < (K_j\tilde{R})^2 < g(x_{max})$ there will exist three positive real solutions.

Because $\omega_j - \Omega > 0$, we arrive at the following condition:

$$\omega_j - \Omega < \frac{\lambda_j}{\sqrt{3}}. \tag{S71}$$

Simulation results show that this condition is not satisfied with our parameters ($\lambda_j = 2$ and $\omega_j \simeq 10 \cdot 2\pi$), when the coupling strength is sufficiently large. Especially as the coupling strength gets larger, $A^2 \ll 3B^2$. Once again, we do not have to rely on the simulation to show that such is the case.

We modify Eq. (S71) slightly:

$$-r_j^{*2} + \sqrt{3}(\omega_j - \Omega) < -r_j^{*2} + \lambda_j. \tag{S72}$$

From Eq. (S62) and Eq. (S61), We can replace the second term of the left side of the Eq. (S71) by $\sqrt{3}K_j\frac{\tilde{R}}{r_j^*}\sin(\phi_j^* - \Phi + \beta)$ and the right side by $K_j\tilde{R}\cos(\phi_j^* - \Phi + \beta)$, respectively:

$$-r_j^{*2} + \sqrt{3}K_j\frac{\tilde{R}}{r_j^*}\sin(\phi_j^* - \Phi + \beta) < -K_j\frac{\tilde{R}}{r_j^*}\cos(\phi_j^* - \Phi + \beta). \tag{S73}$$

Rearranging and using trigonometric identities we arrive at:

$$K_j\frac{\tilde{R}}{r_j^*}\sin(\phi_j^* - \Phi + \beta + \frac{\pi}{6}) < \frac{r_j^{*2}}{2}. \tag{S74}$$

For large K_j , from Eq. (S79) to Eq. (S82) we can write:

$$r_j^* \approx (K_j\tilde{R})^{1/3}. \tag{S75}$$

Putting Eq. (S75) into Eq. (S74), we arrive at

$$\sin(\phi_j^* - \Phi + \beta + \frac{\pi}{6}) < \frac{1}{2}. \tag{S76}$$

Since $(\phi_j^* - \Phi + \beta) \in (0, \pi/2)$, the left side of this inequality is always larger than $\frac{1}{2}$, and therefore Eq. (S76) cannot be satisfied. In the conclusion,the condition for *case ii* cannot be satisfied for large K_j.

Therefore, for large k_j, it is the *case i* the model belong to: $g(x)$ monotonically increasing with x. In this case, as depicted in Illustration 2 (a), as the value of $(K_j\tilde{R})^2$ gets larger, the intersection between $g(x)$ and $(K_j\tilde{R})^2$ will always occur at a higher value of x:

$$x_\varphi > x_\vartheta \quad for \quad K_\varphi > K_\vartheta, \tag{S77}$$

where x_φ and x_ϑ are the solutions to

$$g(x_\varphi) = (K_\varphi\tilde{R})^2 \quad \& \quad g(x_\vartheta) = (K_\vartheta\tilde{R})^2. \tag{S78}$$

Using the definition $x \equiv r_j^{*2}$, we conclude that the amplitude increases as K_j increases.

We can also analyze how fast r_j^* increases as K_j gets larger. We can divide the curve $g(x)$ for $x > 0$ into three ranges, and analyze behavior of the curve within each range: $0 < x < x_{max}$, $x_{max} < x < x_{min}$, and $x_{min} < x$. For each range, we can expand and approximate $g(x)$ by assuming that $g(x)$ is near $x = 0$, near $x = 2A/3$ (the point of inflection where the concavity of the curve changes), and $x \gg 0$ for each range respectively:

$$
\begin{cases}
g(x) \approx (A^2 + B^2)x & for \quad 0 < x < x_{max}, \\
g(x) \approx (-\dfrac{A^2}{3} + B^2)x & for \quad x_{max} < x < x_{min}, \\
g(x) \approx x^3 & for \quad x > x_{min}.
\end{cases}
\tag{S79}
$$

If the intersection of $g(x)$ and $K_j\tilde{R}$ occurs in each range ($g(x) = K_j\tilde{R}$), using the definition $x \equiv r_j^{*2}$, we can write

$$
\begin{cases}
(K_j\tilde{R})^2 \approx (A^2 + B^2)r_j^{*2} & for \quad 0 < x < x_{max}, \\
(K_j\tilde{R})^2 \approx (-\dfrac{A^2}{3} + B^2)r_j^{*2} & for \quad x_{max} < x < x_{min}, \\
(K_j\tilde{R})^2 \approx (r_j^{*2})^3 & for \quad x > x_{min},
\end{cases}
\tag{S80}
$$

or,

$$
\begin{cases}
r_j^* \approx \dfrac{K_j\tilde{R}}{\sqrt{A^2 + B^2}} & for \quad 0 < x < x_{max}, \\
r_j^* \approx \dfrac{K_j\tilde{R}}{\sqrt{-A^2/3 + B^2}} & for \quad x_{max} < x < x_{min}, \\
r_j^* \approx (K_j\tilde{R})^{1/3} & for \quad x > x_{min}.
\end{cases}
\tag{S81}
$$

For small x, $g(x) \sim x$ and for large x, $g(x) \sim x^3$. Also, because the interception of $g(x)$ and K_j is bound to occur at a larger value of x as K_j increases, we can summarize the results as the following:

$$
\begin{aligned}
r_j^* &\approx K_j\tilde{R} \quad & for \quad small \ \ K_j, \\
r_j^* &\approx (K_j\tilde{R})^{1/3} \quad & for \quad large \ \ K_j.
\end{aligned}
\tag{S82}
$$

We match the above results with the Stuart-Landau model on a network,

$$
\dot{z}_j(t) = \{\lambda_j + i\omega_j - |z_j(t)|^2\}z_j(t) + S\sum_{k=1}^{N} A_{jk}z_k e^{i\tau}, \tag{S83}
$$

where S is the coupling strength, and A is the adjacency matrix for the network. The incoming degree (number of connections from other nodes) of node j is denoted as k_j, and A_{jk} is either 1 (there exits a connection from k to j) or 0 (there is

no connection between j and k). We can use the following approximation, if the oscillator connections are not too biased [14, 17]:

$$S\sum_{k=1}^{N} A_{jk} z_k e^{i\tau} \approx \frac{Sk_j}{N}\sum_{k=1}^{N} z_k e^{i\tau}. \tag{S84}$$

Finally,

$$\dot{z}_j(t) = \{\lambda_j + i\omega_j - |z_j(t)|^2\} z_j(t) + \frac{Sk_j}{N}\sum_{k=1}^{N} z_k e^{i\tau}, \qquad j = 1, 2, ..., N, \tag{S85}$$

which is equivalent to Eq. (S52) with $K_j = Sk_j$.

Again, for networks with sufficient connections between different "communities" (i.e., small-world networks), for sufficiently strong coupling strength S such that clusters of entrained oscillators are being formed, the above mean-field approximation approach holds well [17].
Combining the relation $K_j = Sk_j$ with Eq. (S82),

$$\begin{aligned} r_j^* &\sim Sk_j\tilde{R} \quad && for \quad small \;\; Sk_j, \\ r_j^* &\sim (Sk_j\tilde{R})^{1/3} \quad && for \quad large \;\; Sk_j. \end{aligned} \tag{S86}$$

Therefore, higher degree nodes will have larger amplitudes, and lower degree nodes will have smaller amplitude.

Before we proceed to calculate the dPLI, we return to Eq. (S61) and (S62). The necessary conditions for the Eq. (S61) and (S62) to have a solution $(r_j^*,\ \phi_j^*)$ are

$$K_j\tilde{R} \geq \{r_j^{*2} - \lambda_j\} r_j^*, \tag{S87}$$

$$K_j\tilde{R} \geq \{\omega_j - \Omega\} r_j^*. \tag{S88}$$

For the Stuart-Landau model on a network, we can write

$$Sk_j\tilde{R} \geq \{r_j^{*2} - \lambda_j\} r_j^*, \tag{S89}$$

$$Sk_j\tilde{R} \geq \{\omega_j - \Omega\} r_j^*. \tag{S90}$$

If the strength S is small enough, or k_j is small, then there may be oscillators not able to have a stationary solution and drift just as in the case of the Kuramoto model. However, for large value of S, as the strength S increases, r_j^* only increases as much as $(K_j\tilde{R})^{1/3}$. Therefore, when we increase value of S, all oscillators will eventually satisfy the necessary conditions sooner, and will asymptotically reach the phase locked solution $(r_j^*,\ \phi_j^*)$ faster. The difference compared to the Kuramoto model comes from the factor r_j^*.

We can further inspect the stability conditions for the solution $(r_j^*,\ \phi_j^*)$:

$$3r_j^{*2} - \lambda_j > 0, \tag{S91}$$

$$\cos(\phi_j^* - \Phi + \beta) > 0. \tag{S92}$$

With Eq. (S61), (S92), we find that $r_j^{*2} - \lambda_j > 0$. This finding will be utilized in the calculation of the dPLI for the Stuart-Landau model.

Directed phase lag index of Stuart-Landau model on complex networks

Eq. (S62) and (S61) can be combined to obtain information about the phase of the oscillators. These equations can be written in the following fashion:

$$-\frac{\lambda_j - r_j^{*2}}{K_j\tilde{R}} r_j^* = \cos(\phi_j^* - \Phi + \beta), \tag{S93}$$

$$\frac{\omega_j - \Omega}{K_j\tilde{R}} r_j^* = \sin(\phi_j^* - \Phi + \beta). \tag{S94}$$

If we divide Eq. (S94) by Eq. (S93), we arrive at

$$\tan(\phi_j^* - \Phi + \beta) = \frac{\omega_j - \Omega}{r_j^{*2} - \lambda_j}. \tag{S95}$$

If $r_\varphi^* > r_\vartheta^*$, assuming $\lambda_\varphi^* = \lambda_\vartheta^* = \lambda$, then $(\omega_\varphi - \Omega)/(r_\varphi^{*2} - \lambda) < (\omega_\vartheta - \Omega)/(r_\vartheta^{*2} - \lambda)$. Again assuming $\omega_\varphi = \omega_\vartheta = \omega$, since $\omega - \Omega > 0$ and $r_j^{*2} - \lambda > 0$ for any j ,

$$\tan(\phi_\varphi^* - \Phi + \beta) < \tan(\phi_\vartheta^* - \Phi + \beta). \tag{S96}$$

If $(\phi_j^* - \Phi + \beta) \in [-\pi/2, \pi/2]$ (which is confirmed in the previous section), the following holds:

$$If \quad r^*_\varphi > r^*_\vartheta \quad then \quad \phi^*_\varphi < \phi^*_\vartheta. \tag{S97}$$

To summarize, nodes with larger amplitude will will phase-lag compared to nodes with smaller amplitudes.

From Eq. (S82) and (S86), we know that the amplitude is proportional to the degree of the node. Therefore, higher degree nodes will phase-lag, whereas lower degree nodes phase-lead.

Our simulation results are shown in S4 Figure, and confirm our analytic results. Again, as in the case of the Kuramoto model, we assumed constant time delay τ and therefore constant phase delay β between oscillators in our derivations, and assumed that the given natural frequencies of the oscillators are all equal to each other: $\omega_j = \omega$. In the simulations on Gong's anatomic human brain network, both distance-dependent time delay and constant time delay were applied, both showing the negative correlation between the node degree and dPLI as long as the delays are smaller than a quarter of one oscillating cycle. For Fig. 4 in the main text where distance-dependent time delay was used, Spearman correlation coefficient was -0.61, whereas constant time delay was used, the coefficient was -0.63 both with $p < 0.01$. The natural frequencies of the oscillators were given as a Gaussian distribution with mean at 10 Hz and standard deviation 1 (making ω_j around 10·2π rad/s) in simulations. The results were similar with the case where all natural frequencies were equal ($\omega_j = \omega$). The variations in the time delay τ and the natural frequency ω_j act as perturbations to each oscillators while maintaining the overall tendency of the negative correlation. In the simulations a Gaussian white noise $\xi(t)_j$ of vanishing mean and standard deviation of 2 was added to each oscillator's equation, to test the robustness of our results against the noise. As shown in S4 Figure the main finding of the analysis was still maintained with these perturbations. Higher degree nodes phase-lag, whereas lower degree nodes phase-lead. We also supplement our results with S2 Figure highlighting the distinct local dynamics for the hub nodes and the periphery nodes, regardless of network type (scale-free network, random network, hierarchical modular network [25], and brain networks of Gong and Hagmann).

4 Comparison between different measures for the Stuart-Landau model

As mentioned in section 2, dPLI reflects which of two signals is leading and which is lagging in phase. This phase-lead/lag relationship is used as a surrogate for the direction of information flow. It can be asserted that all causal influences lead and resultant effects lag, simply by virtue of the temporal constraints on cause-effect relationships. However, the converse assertion that every lead/lag relationship reflects a causal influence does not hold. As such, we conducted parallel analyses with the causality measure Granger causality (GC) and the information-theoretic measure symbolic transfer entropy (STE).

GC is a statistical concept of causality based on whether one time series is useful in predicting another. A signal X "Granger-causes" another signal Y, if past values of X provide statistically significant information in predicting Y, more so than the information contained in the past values of Y alone [26, 27].

Transfer Entropy is an analytic technique rooted in information theory, and is a surrogate for the transfer of information between two signals. Transfer Entropy from a signal X to another signal Y is the amount of uncertainty reduced in future values of Y, by knowing the past values of X given past values of Y. STE is a simplified version of the Transfer Entropy, that uses the technique of symbolization [28, 29]. Here we used STE to utilize its robustness and computationally faster speed.

dPLI, GC, and STE were applied to the Stuart-Landau model on Gong's human brain network with distance dependent time delays between nodes(S1 Figure). The results with constant time delay was also similar. For GC, Seth's toolbox was used to compute [27]. The results show qualitatively similar findings across dPLI, GC, and STE, with quantitative differences relating to the coupling strength at which the phenomenon is clearly manifest. All results show that the nodes with higher degree either phase-lag (dPLI: S1 Figure (A)), Granger-caused (GC: S1 Figure (B)), or information transferred (STE: S1 Figure (C)). Negative correlations between node degree, and the measures dPLI, GC and STE are apparent (average Spearman correlation coefficients of -0.60, -0.59 and -0.54 respectively, with $P<0.01$ for the coupling strength of $1.5 \sim 10$ for dPLI and GC, and $15 \sim 50$ for STE). The results suggest that the phase-lead/lag relation, causality, and the information flow transfer are possibly all correlated with each other, supporting the general interpretation that dPLI reflects information transfer despite the fact that it is not a direct measure of information transfer.

Supporting References

[1] Hoppensteadt FC, Izhikevich EM. Weakly Connected Neural Networks. 1st ed. New York: Springer-Verlag; 1997.

[2] Pikovsky A, Rosenblum M, Kurths J. Synchronization. 1st ed. New York: Cambridge University Press; 2001.

[3] Bonnin M, Corinto F, Gilli M. Phase model reduction and phase locking of coupled nonlinear oscillators. Int J bif and Chaos. 2010;20: 645-656.

[4] Guckenheimer J, Holmes P. Nonlinear Oscillations, Dynamical Systems, and Bifurcations of Vector Fields. 1st ed. New York: Springer-Verlag; 1983.

[5] Strogatz SH. Nonlinear Dynamics and Chaos. 1st ed. Cambridge (MA): Westview Press; 1994.

[6] Kuramoto Y. International Symposium on Mathematical Problems in Theoretical Physics, Lecture Notes in Physics, Vol. 39. 1st ed. Arakai H, editor. New York: Springer; 1975.

[7] Strogatz SH. From Kuramoto to Crawford: exploring the onset of synchronization in populations of coupled oscillators. Physica D. 2000;143: 1-20.

[8] Wilson HR, Cowan JD. Excitatory and inhibitory interactions in localized populations of model neurons. Biophys J. 1972;12: 1-24.

[9] Destexhe A, Sejnowski TJ. The Wilson–Cowan model, 36 years later. Biol Cybern. 2009;101: 1-2.

[10] Schuster HG, Wagner P. A model for neuronal oscillations in the visual cortex: 1. Mean-field theory and derivation of the phase equations. Biol Cybern. 1990;64: 77-82.

[11] Schuster HG, Wagner P. A model for neuronal oscillations in the visual cortex: 2. Phase description of the feature dependent synchronization. Biol Cybern 1990;64: 83-85.

[12] Daffertshofer A, van Wijk BCM. On the influence of amplitude on the connectivity between phases. Front Neuroinformatics. 2011;5: 8.

[13] Albert R, Barabási A-L. Statistical mechanics of complex networks. Rev Mod Phys. 2002;74: 47.

[14] Ko TW, Ermentrout GB. Partially locked states in coupled oscillators due to inhomogeneous coupling. Phys Rev E. 2008;78: 016203.

[15] Abrams DM, Strogatz SH. Chimera states in a ring of nonlocally coupled oscillators. Int J bif and Chaos. 2006;16: 21-37.

[16] Izhikevich EM. Phase models with explicit time delays. Phys Rev E. 1998;58: 905.

[17] Hong H, Park H, Tang L-H. Finite-size scaling of synchronized oscillation on complex networks. Phys Rev E. 2007;76: 066104.

[18] Stam CJ, Nolte G, Daffertshofer A. Phase lag index: assessment of functional connectivity from multi channel EEG and MEG with diminished bias from common sources. Hum Brain Mapp. 2007;28: 1178-1193.

[19] Stam CJ, van Straaten ECW. Go with the flow: use of a directed phase lag index (dPLI) to characterize patterns of phase relations in a large-scale model of brain dynamics. NeuroImage. 2012;62: 1415-1428.

[20] Selivanov AA, Lehnert J, Dahms T, Hövel P, Fradkov AL, Sch öll E. Adaptive synchronization in delay-coupled networks of Stuart-Landau oscillators. Phys Rev E. 2012;85: 016201.

[21] Bergner A, Frasca M, Sciuto G, Buscarino A, Ngamga EJ, Fortuna L, Kurths J. Remote synchronization in star networks. Phys Rev E. 2012;85: 026208.

[22] Gambaudo JM. Perturbation of a Hopf bifurcation by an external time-periodic forcing. J Differential Equations. 1985;57: 172-199.

[23] Marques F, Meseguer A, Lopez JM, Pacheco JR, Lopez JM. Bifurcations with imperfect SO(2) symmetry and pinning of rotating waves. Proc R Soc A. 2013;469: 20120348.

[24] Le Gal P, Nadim A, Thompson M. Hysteresis in the forced Stuart-Landau equation: application to vortex shedding from an oscillating cylinder. J Fluids and Structures. 2001;15: 445-457.

[25] Sales-Pardo M, GuimeraŔ, Moreira A, Amaral L. Extracting the hierarchical organization of complex networks. Proc Natl Acad Sci USA. 2007;104: 15224-15229.

[26] Granger CWJ. Investigating causal relations by econometric models and cross-spectral methods. Econometrica. 1969;37: 424-438.

[27] Seth AK. A MATLAB toolbox for Granger causal connectivity analysis. J Neurosci Methods. 2010;186: 262-273.

[28] Staniek M, Lehnertz K. Symbolic transfer entropy. Phys Rev Lett. 2008;100: 158101.

[29] Lee U, Ku S, Noh G-J, Baek S, Choi B-M, Mashour GA. Disruption of frontal–parietal communication by Ketamine, Propofol, and Sevoflurane. Anesthesiology. 2013;118: 1264-1275.
